# Outcomes of Patients With Primary Cardiac Diagnoses Admitted to Cardiac vs Noncardiac Intensive Care Units

**DOI:** 10.1016/j.jacadv.2022.100114

**Published:** 2022-10-28

**Authors:** Samuel B. Brusca, Panagis Galiatsatos, Sarah Warner, Xiaobai Li, Tiffany M. Powell-Wiley, Sameer S. Kadri, Michael A. Solomon

**Affiliations:** a Critical Care Medicine Department, National Institutes of Health Clinical Center, Bethesda, Maryland, USA; b Division of Cardiology, Department of Internal Medicine, University of California, San Francisco, California, USA; c Division of Pulmonary and Critical Care Medicine, The Johns Hopkins School of Medicine, Baltimore, Maryland, USA; d Biostatistics and Epidemiology Service, National Institutes of Health Clinical Center, Bethesda, Maryland, USA; e Cardiovascular Branch, National Heart, Lung, and Blood Institute, National Institutes of Health, Bethesda, Maryland, USA; f Intramural Research Program, National Institute on Minority Health and Health Disparities, National Institutes of Health, Bethesda, Maryland, USA.

**Keywords:** cardiac intensive care, cardiogenic shock, critical care cardiology, noncardiac critical illness, noncardiac ICU, staffing models

## Abstract

**BACKGROUND:**

Demographics in cardiac intensive care units (CICUs) have evolved, with increased prevalence of noncardiac critical illnesses.

**OBJECTIVES:**

This study compares outcomes of patients with primary cardiac diagnoses admitted to CICUs vs those of patients with primary cardiac diagnoses admitted to noncardiac ICUs.

**METHODS:**

The Cerner Health Facts Database was queried to identify adults with primary cardiac diagnoses admitted to ICUs within 48 hours of presentation between 2009 and 2014. Only hospitals with multiple ICUs including a CICU were studied. Information on ICU staffing was not available. A univariate analysis of ICU type (model 1) and multivariate analyses incorporating patient- and hospital-level variables (model 2) and concurrent, noncardiac, ICU-level diagnoses (model 3) were utilized to assess the impact of ICU type on inpatient mortality.

**RESULTS:**

Of 16,163 encounters across 14 hospitals, 8,499 (52.6%) were admitted to CICUs and 7,664 (47.4%) to noncardiac ICUs. Univariate analysis (model 1) demonstrated increased mortality in noncardiac ICUs compared to CICUs (odds ratio [OR]: 1.47, 95% CI: 1.32–1.64; *P* < 0.0001). This risk dissipated (OR: 1.04, 95% CI: 0.91–1.18; *P* = 0.56) after controlling for patient- and hospital-level variables (model 2). Inclusion of concurrent, noncardiac, ICU-level diagnoses (model 3) lead to a reversal with decreased mortality in noncardiac ICUs (OR: 0.86, 95% CI: 0.76–0.98; *P* = 0.03).

**CONCLUSIONS:**

In this historical cohort study evaluating CICU outcomes prior to the evolution of proposed staffing and care model modernization, survival of cardiac patients with concurrent, noncardiac critical illnesses may have been better with the expertise available in general system ICUs. These results may support contemporary efforts to increase the capacity to manage noncardiac critical illnesses in CICUs.

First instituted in the early 1960s, dedicated specialty units devoted to cardiac disease have had far-reaching effects on the landscape of cardiovascular care.^1^ Initially described as coronary care units (CCUs), these wards were created to decrease inpatient mortality associated with acute coronary syndrome (ACS). To this end, CCUs were quite successful, with a uniquely trained staff of physicians and nurses heralding improved patient outcomes that persisted through the reperfusion era.^2,3^ Over time, however, CCUs have evolved into cardiac intensive care units (CICUs), where patients with primary cardiac pathology are treated for a range of diverse diseases, frequently complicated by multiorgan dysfunction.^4–11^ Recently, a critical care cardiology registry identified respiratory failure as the most frequent indication for ICU-level care in CICU patients.^8^ Although treatment of ACS remains a staple of the CICU, there has been a cultural shift, with some even questioning whether routine care of ACS warrants CICU admission.^12^ Furthermore, with improved recognition and time to revascularization, acute myocardial infarction complicated by shock may represent a dwindling CICU demographic.^9^

Increasing patient complexity has led to increasing utilization of mechanical circulatory support and other high-intensity therapies such as mechanical ventilation. It also prompted investigations into the importance of critical care medicine training in the CICU, with studies demonstrating improved patient outcomes.^13–17^ This may be due to the increasing frequency of noncardiac illness in the CICU and its significant role as a driver of CICU patient mortality.^4,7,10,18^ Recent data have highlighted the impact of renal failure, systemic inflammation/infection, and mechanical ventilation for respiratory failure on CICU outcomes.^19–21^ Despite these findings, adoption of staffing models that incorporate dedicated critical care cardiologists or critical care medicine comanagement teams has been slow. As of 2017, the majority of CICUs remained traditionally staffed units without dedicated critical care cardiologists, even among academic referral centers.^22^ Additionally, despite the clinical relevance of an expanded critical care medicine skill set, there remains only a small number of providers with dual certification in cardiology and critical care medicine.^15^

Given the apparent mismatch between evolving CICU patient demographics and staffing models, which may deprive cardiac patients of essential critical care expertise, we sought to investigate the role of ICU type on cardiac patient outcomes. We leveraged a large multicenter electronic health record repository to better discern the short-term prognosis of patients with primary cardiac diagnoses when admitted to CICUs as compared to admission to noncardiac ICUs.

## METHODS

### STUDY DESIGN.

**FIGURE 1:**
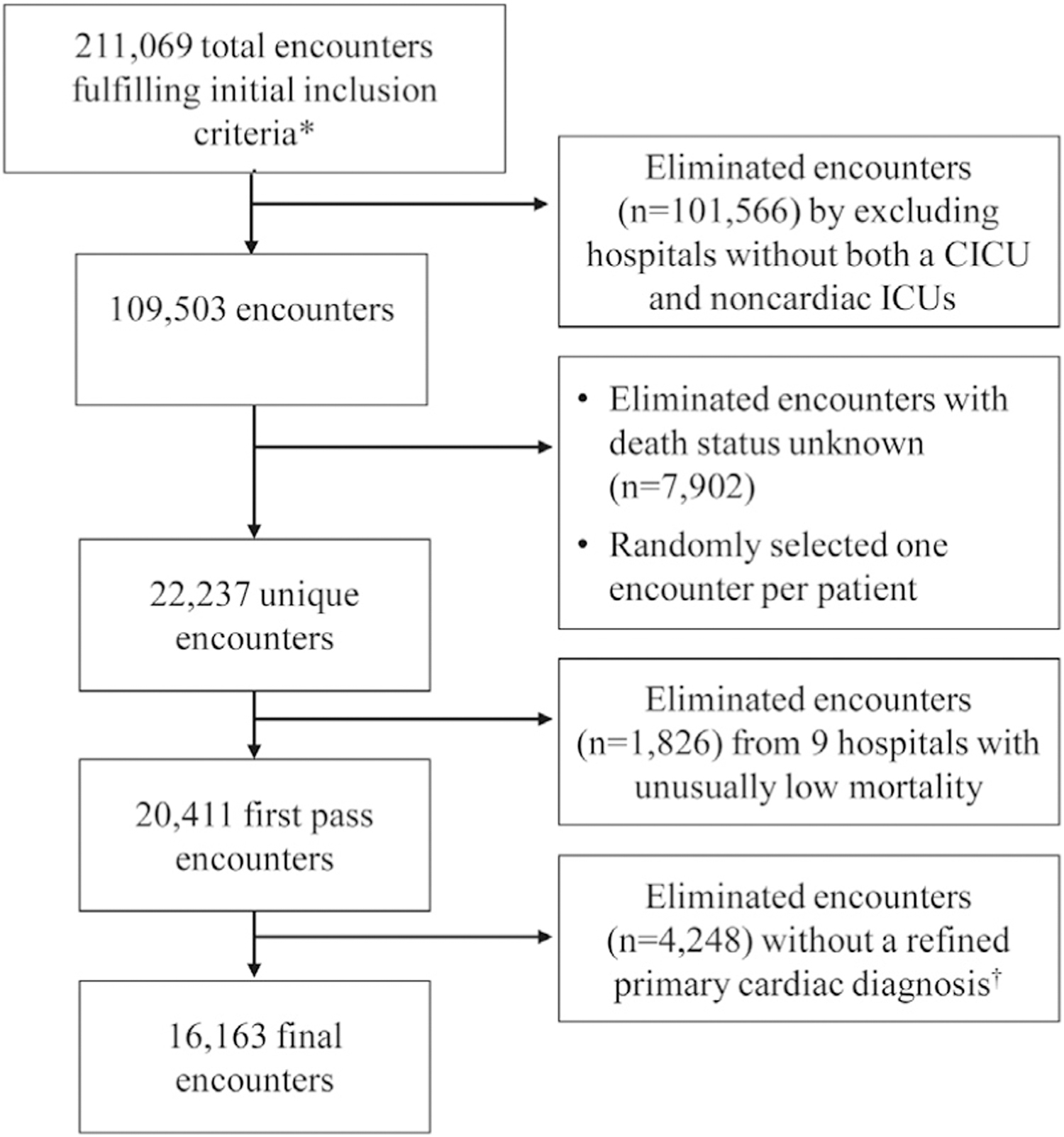
Flow Chart Delineating Encounter Selection From the Cerner Health Facts Database A total of 211,069 encounters were initially selected from over 6 million encounters based on the following inclusion criteria (*): admission year 2009 to 2014, age ≥20 years, the presence of a primary cardiac diagnosis code from the initial diagnoses list (ICD-9 codes in Supplemental Table 1), recorded ICU stay within 48 hours of admission, only 1 ICU stay for the given encounter, at least 1 time-stamped procedure during the ICU stay. At least 1 medication order and 1 lab order during the encounter was also required. The 211,069 encounters were further paired to only include hospitals with both a CICU and non-CICU. Encounters with unknown survival status were eliminated, and 1 encounter was selected at random per patient. Nine additional hospitals were considered outliers based on unusually low or no deaths. Finally, encounters were further limited to those with a more selective set of primary cardiac diagnoses (†) (ICD-9 codes in Supplemental Table 2). CICU = cardiac intensive care unit; ICD-9 = International Classification of Diseases-9th Revision.

The National Institutes of Health Office of Human Subjects Research waived institutional review board evaluation since analyses were restricted to deidentified data. This retrospective cohort study utilized electronic health records gathered from the Cerner Health Facts Database. The Cerner Health Facts Database incorporates deidentified patient clinical data from a large number of geographically diverse US hospitals and has been used for mortality analyses in sepsis and heart failure.^23,24^ The database was queried to identify a sample of adult patients (aged 20 years and older) hospitalized from January 1, 2009, to December 31, 2014. Inpatient encounters were selected if they recorded a primary diagnosis from a broad range of preselected cardiac diagnoses and displayed admission to the ICU or transfer to the ICU within 48 hours of presentation. The sample was secondarily limited to: 1) patients with a more specific set of cardiac diagnoses (eg, excluding inpatient encounters with a primary diagnosis of type II diabetes mellitus, a risk factor of ischemic heart disease but not directly a cardiac diagnosis); and 2) patients admitted to hospitals with multiple adult ICUs, including at least 1 ICU identified as a CCU or CICU (Figure 1). For outcome analyses, cardiac patients were dichotomized based on admission to CICUs vs noncardiac ICUs (medical, surgical, and general [undefined] ICUs).

Data for the study were restricted to the period preceding universal implementation of International Classification of Diseases Tenth Revision, Clinical Modification. Thus, patients with primary cardiac diagnoses were identified utilizing International Classification of Diseases Ninth Revision (ICD-9), Clinical Modification, codes listed for hospitalization.^25^ As part of data cleaning, an initial set of less-specific diagnoses (Supplemental Table 1) was trimmed to a more representative set of cardiac diagnoses prior to analysis (Supplemental Table 2). The cardiac diagnosis was identified as the primary hospital diagnosis when it was adjudicated as the priority-one diagnosis code for the encounter in Cerner Health Facts (equivalent to “principal diagnosis code” in discharge abstracts). These cardiac diagnoses were also grouped into 9 categories including ACS/coronary artery disease (CAD), heart failure/shock, cardiac arrest/arrhythmia, valvular disease, peripheral arterial disease/aortic disease, periprocedural monitoring, no acute cardiovascular disease (eg, primary chronic cardiac condition with undefined ICU indication), other cardiac disease, and multiple cardiac diagnoses (Supplemental Table 2), which a recent study has shown represents diagnosis categories frequently seen in CICUs.^8^ Additional demographic and clinical data were also collected, including coded comorbid conditions, admission Sequential Organ Failure Assessment (SOFA) scores, total hospital and ICU lengths of stay, as well as coded noncardiac diagnoses that typically warrant ICU admission (eg, sepsis and respiratory failure). Coded comorbid conditions were used to calculate the Elixhauser Comorbidity Index for each patient.^26^ Concurrent noncardiac diagnoses displaying a high probability for requiring ICU admission were adapted from those previously reported in the Multiparameter Intelligent Monitoring in Intensive Care II database and further grouped into organ-system categories for comparison between groups (Supplemental Table 3).^27^ The primary outcome was death in hospital or discharge to hospice.

Hospital-level variables, including location (geographic census region and urban vs rural), bed size, annual admissions, and academic teaching status, were recorded. However, hospital-level variables for ICU staffing and care models were not available. Additionally, hospitals were assigned a level status indicating the general complexity of cardiovascular care offered in the CICU identified based on recorded ICD-9 procedure codes. Hospitals performing extracorporeal membrane oxygenation or ventricular assist device implantation were designated level I. Hospitals without both cardiac catheterization labs and cardiovascular surgery programs were designated level III, and all other hospitals were level II.^5^

### STATISTICAL ANALYSIS.

Summary demographic variables and clinical data for patients in both groups (cardiac patients admitted to CICUs and cardiac patients admitted to noncardiac ICUs) were collected (Table 1). Hospital-level variables were also tabulated (Table 2). An initial unadjusted logistic regression was performed to compare mortality rates between CICU and noncardiac CICU patients at each individual hospital. Multilevel models were constructed, taking into consideration the correlations and clustered nature of the patients’ data within hospitals and utilizing secondary covariates that investigators hypothesized would impact ICU outcomes and/or modify the potential benefit of CICU admission. A univariate multilevel model (model 1) including care in a CICU vs noncardiac ICU as the only fixed effect and treating hospital specification as a random effect was first constructed. This was followed by 2 additional multilevel models. In addition to CICU vs noncardiac ICU admission status, model 2 also incorporated patient demographics (eg, age, sex), patient clinical data (eg, cardiac diagnosis type, Elixhauser comorbidity score, SOFA score), and hospital-level variables (eg, rural vs urban, bed number, teaching status). Model 3 included all variables in model 2 with the addition of concurrent noncardiac coded diagnoses typically warranting ICU admission grouped by disease category (eg, infectious diseases, respiratory, neurologic). As these other concurrent ICU-level diagnoses were not time-stamped, it is unclear whether they were or were not present on admission (present-on-admission flags in this data set were not deemed reliable upon quality control checks), but either way, they complicated the primary cardiac diagnosis. A full list of variables included in model 2 is presented in Table 3, and for model 3, in Supplemental Table 4. The presented *P* values represent odds ratios (ORs) of inpatient mortality for each individual variable, adjusting for covariates in multivariate models. Subgroup analyses were performed on patients requiring vasopressors, mechanical ventilation, those likely to require renal replacement therapy, and those with concurrent infectious disease diagnoses. Additional subgroup analyses were performed as sensitivity analyses on patients not requiring vasopressors or mechanical ventilation as well as patients within specific cardiovascular diagnosis categories (ACS/CAD, heart failure/shock, cardiac arrest/arrhythmia). Patients requiring vasopressors and mechanical ventilation were identified based on vasopressor orders and ICD-9 procedure codes for mechanical ventilation, respectively. Patients likely to require renal replacement therapy were identified based on creatinine levels (≥5.0). Patients with concurrent infectious disease diagnoses were identified based on the presence of a Multiparameter Intelligent Monitoring in Intensive Care II diagnosis within the infectious disease category (Supplemental Table 3). All statistical analyses were performed in SAS (version 9.4, SAS Institute).

## RESULTS

A total of 16,163 patient encounters with primary cardiac diagnoses were identified at 14 hospitals between January 1, 2009, and December 31, 2014. Encounters were grouped based on ICU admission type, with 8,499 (52.6%) admitted to CICUs (CICU group) and 7,664 (47.4%) admitted to noncardiac ICUs (non-CICU group). Most patients admitted to non-CICUs were cared for in General ICUs (n = 4,631, 60.4%) followed by Medical ICUs (n = 1,981, 25.8%) and surgical ICUs (n = 1,052, 13.7%). Patient demographics, categorization of primary cardiac diagnoses, and the presence of additional diagnoses typically warranting ICU admission are presented in Table 1. The demographic characteristics of the patients at base-line were balanced between the 2 groups, except for small differences. Notably, patients admitted to CICUs compared to non-CICUs had lower mean Elixhauser scores [2.8 ± 2.4 vs 3.2 ± 2.5; *P* < 0.0001] and mean SOFA scores [2.4 ± 3.1 vs 3.7 ± 3.5; *P* < 0.0001].

Of the 14 included hospitals, 12 were level I centers, and 2 were level II centers (Table 2). No level III centers met inclusion criteria given that multi-ICU hospitals generally have in-house cardiac catheterization capability. The hospitals were also predominantly urban with 200 to 499 beds. The geographic distribution was evenly spread between the Northeast, South, and Midwest, with only 1 hospital from the West region.

**FIGURE 2:**
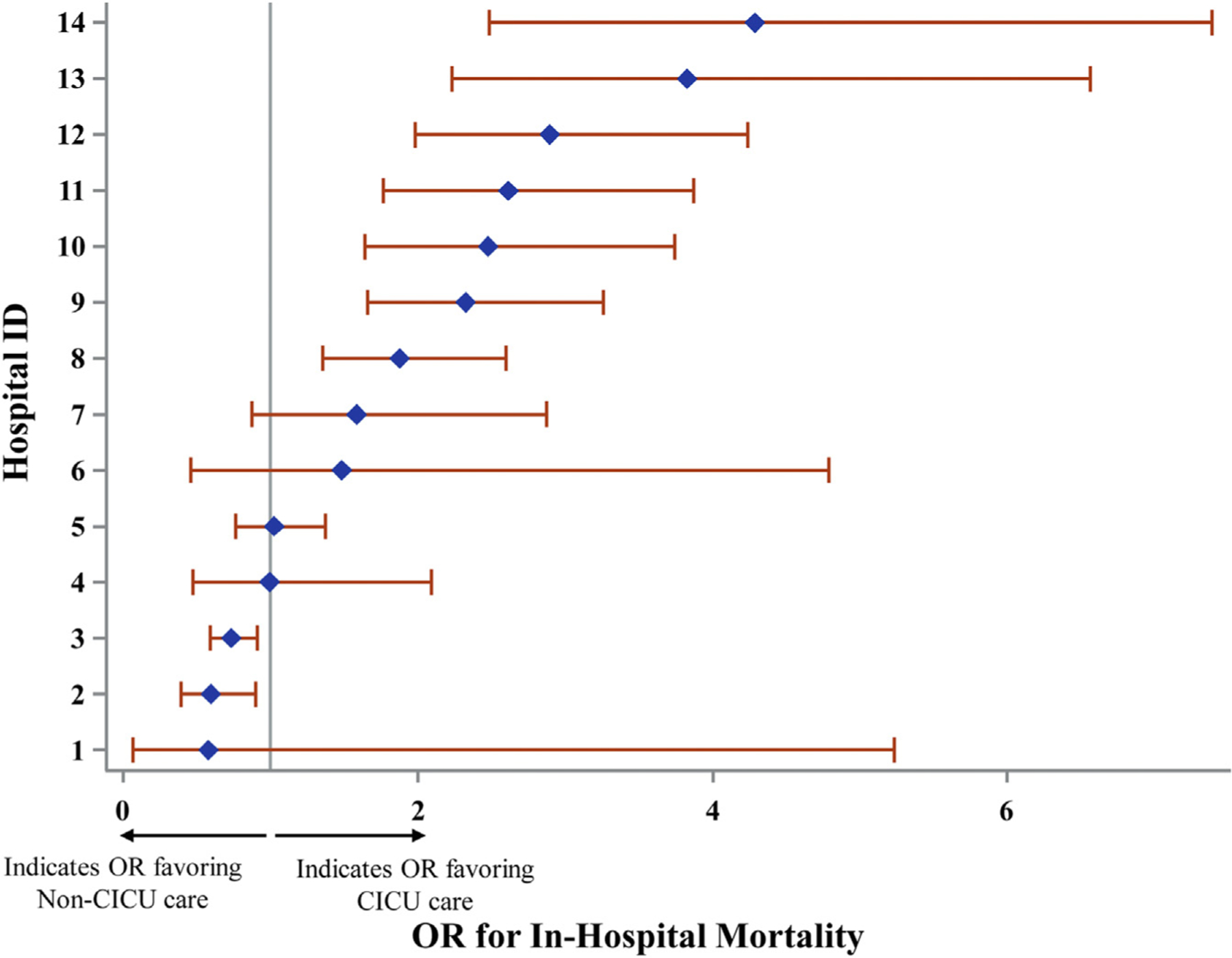
In-Hospital Mortality for Patients With Primary Cardiac Diagnoses Admitted to CICUs vs Noncardiac ICUs at 14 U.S. Centers Displayed are the odds ratios (OR) and 95% CIs for inpatient mortality at each hospital (ID 1 through 14). The **grey vertical line** represents an OR of 1. OR to the **right of the grey line** indicate increased mortality in the non-CICU group whereas OR to the **left of the grey line** indicate decreased mortality in the non-CICU group. Seven hospitals demonstrated statistically significant increased mortality in the non-CICU group, while 2 hospitals demonstrated statistically significant decreased mortality in the non-CICU group. Overall, the OR favored CICU care. CICU = cardiac intensive care unit; ID = identifier.

Utilizing an unadjusted logistic regression model, significantly increased mortality was identified in the non-CICU group compared to the CICU group in 7 of the 14 hospitals, with OR ranging from 1.87 (95% CI: 1.35–2.60) to 4.29 (95% CI: 2.49–7.39). Of the remaining 7 hospitals, 5 demonstrated no significant difference in outcomes, and 2 demonstrated decreased mortality in the non-CICU group, with OR of 0.59 (95% CI: 0.39–0.90) and 0.73 (95% CI: 0.59–0.91), respectively (Figure 2). Next, multilevel modeling was performed starting with a univariate analysis (model 1) comparing CICU vs non-CICU groups. Consistent with the logistic regression, increased mortality was identified in the non-CICU group (Central Illustration) (OR: 1.47, 95% CI: 1.32–1.64; *P* < 0.0001). Subsequently, a multivariate model (model 2; Table 3), incorporating patient- and hospital-level variables, was performed. As expected, patient variables such as increasing patient age (OR: 1.03, 95% CI: 1.03–1.04; *P* < 0.0001), SOFA score on admission (OR: 1.32, 95% CI: 1.30–1.34; *P* < 0.0001), and Elixhauser Comorbidity Index (OR: 1.09, 95% CI: 1.06–1.12; *P* < 0.0001) were associated with increased mortality. There were no mortality differences based on race; however, there was increased mortality for those patients with non-Medicare insurance coverage (Medicaid, self-insured, unspecified) compared to patients with Medicare (see Table 3 for individual ORs; *P* = 0.001). Male sex (OR: 0.79, 95% CI: 0.71–0.89; *P* < 0.0001) and admission to an urban hospital (OR: 0.31, 95% CI: 0.13–0.73; *P* < 0.01) were associated with decreased mortality. The model also incorporated cardiac diagnosis type to account for potential discrepancies in the distribution of cardiac illnesses between the CICU and non-CICU groups. Cardiac diagnosis type also had a significant effect on patient outcomes, with cardiac arrest/arrythmia, heart failure/shock, and “other” (largely composed of congenital and pericardial heart diseases) demonstrating the highest mortality (see Table 3 for individual ORs; *P* < 0.0001). Importantly, after accounting for these additional variables, patients with primary cardiac diagnoses admitted to a non-CICU as opposed to a CICU no longer demonstrated increased mortality (Central Illustration) (OR: 1.04, 95% CI: 0.91–1.18; *P* = 0.56). Finally, a third model (model 3; Supplemental Table 4) was constructed, incorporating all the patient- and hospital-level variables previously included in model 2 as well as the presence of concurrent noncardiac diagnoses typically warranting ICU admission grouped by organ system. Variables including age, SOFA score, and non-Medicare insurance remained predictive of increased mortality. Cardiac arrest/arrythmia, heart failure/shock, and “other” continued to demonstrate the highest mortality. Similarly, male sex and urban hospital setting remained associated with decreased mortality (Supplemental Table 4). Of the newly included variables, noncardiac ICU-level diagnoses in the categories of respiratory disease, infectious disease, gastrointestinal disease, and neurologic disease were also associated with increased mortality. Importantly, with the addition of these new concurrently present ICU-level noncardiac diagnoses, admission to a non-CICU as compared to a CICU resulted in statistically significant decreased mortality (Central Illustration) (OR: 0.86, 95% CI: 0.76–0.98; *P* = 0.03).

In order to identify specific patient populations that could be driving the association between non-CICU admission and improved outcomes, subgroup analyses were performed on patients requiring vasopressors and mechanical ventilation, patients with a high probability of requiring renal replacement therapy, and patients with concurrent infectious disease diagnoses. Examination of only patients requiring vasopressors (n = 3,751) revealed decreased mortality in the non-CICU group compared to the CICU group in all 3 models (model 1: OR: 0.71, 95% CI: 0.59–0.86, *P* < 0.001; model 2: OR: 0.61, 95% CI: 0.50–0.76, *P* < 0.0001; model 3: OR: 0.58, 95% CI: 0.46–0.72, *P* < 0.0001). Similarly, patients requiring mechanical ventilation (n = 817) admitted to non-CICUs also had improved outcomes across all models compared to those admitted to CICUs (model 1: OR: 0.50, 95% CI: 0.36–0.68, *P* < 0001; model 2: OR: 0.54, 95% CI: 0.36–0.80, *P* < 0.01; model 3: OR: 0.55, 95% CI: 0.37–0.82, *P* < 0.01). In contrast, patients with a high probability of requiring renal replacement therapy (creatinine ≥5.0; n = 561) had similar mortality in both univariate and multivariate modeling (model 1: OR: 1.23, 95% CI: 0.76–2.00; model 2: OR: 0.99, 95% CI: 0.56–1.75; model 3: OR: 0.94, 95% CI: 0.52–1.72). There were also no differences in patients with concurrent, ICU-level infectious disease diagnoses (model 3: OR: 1.14, 95% CI: 0.91–1.42).

Lastly, additional subgroups were investigated in sensitivity analyses, limiting the cohort to patient groups less likely to benefit from non-CICU admission. These included patients not requiring mechanical ventilation or vasopressors as well as patients within specific cardiovascular disease categories (heart failure/shock, CAD/ACS, and arrhythmia/arrest). Taken together (Supplemental Table 5), these analyses demonstrated a potential benefit for CICU admission compared to non-CICU admission in model 2; however, significance largely dissipated in model 3. This held true for patients (n = 12,141) not requiring mechanical ventilation or vasopressors (model 2: OR: 1.4, 95% CI: 1.20–1.64; model 3: OR: 1.10, 95% CI: 0.94–1.30) as well as patients (n = 1,057) with heart failure/shock not requiring mechanical ventilation or vasopressors (model 2: OR: 1.67, 95% CI: 1.13–2.48; model 3: OR: 1.36, 95% CI: 0.9–2.06; additional sensitivity analysis results in Supplemental Table 5).

## DISCUSSION

A large multihospital database was used to identify patients with primary cardiac diseases warranting ICU admission. Initially, the analysis identified a 47% increased risk of mortality when cardiac patients were triaged to a non-CICU. However, after accounting for other patient- and hospital-level variables, this benefit dissipated. Furthermore, when concurrent noncardiac ICU-level diagnoses were added to the model, there was a reversal in the mortality trend, with patients admitted to a noncardiac ICU faring significantly better than those admitted to a CICU (14% risk reduction). Subgroup analyses performed to identify patients that may benefit most from non-CICU admission demonstrated consistently decreased mortality in the non-CICU group for patients requiring vasopressors and mechanical ventilation. In contrast, analyses performed on patients likely to have received renal replacement therapy and those with concurrent infectious disease diagnoses demonstrated no differences between CICU and non-CICU groups. Finally, sensitivity analyses investigating patients less likely to benefit from non-CICU care, including those not requiring mechanical ventilation or vasopressors, showed that these patients appeared to have lower mortality in CICUs in model 2, but this significance dissipated in model 3 (includes concurrent noncardiac ICU-level diagnoses).

Together, these results imply that the potential benefit of cardiac specialty care for cardiac patients admitted to the CICU may be counteracted by the presence of complex noncardiac ICU-level diagnoses such as multiorgan failure. One interpretation is that primary cardiac patients with multiorgan failure are critically ill to the point that more than cardiac-specific care experience is needed to preserve meaningful benefit from CICU admission. Furthermore, many CICUs continue to be staffed by cardiologists without critical care training, and these providers may be less facile with the management of general critical care issues. Only recently has staffing of CICUs by critical care-trained cardiologists become more sought after. However, the total number of dual-trained physicians in cardiology and critical care medicine remains insufficient, and the majority of units remain traditionally staffed.^5,15,22,28^

Subgroup analyses implicated that patients with respiratory failure requiring mechanical ventilation and those with shock requiring vasopressors fared better in non-CICUs than in CICUs, while outcomes among patients with infections and those with imminent renal failure were similar across care settings. These findings may be explained by inherent differences in the clinical expertise, patient acuity, and associated clinical support that these diagnoses entail. The management of vasopressors and mechanical ventilation are some of the most dynamic processes occurring in critical care, likely requiring and benefiting from immediately available and ongoing expertise in critical care medicine and cardiology. Both renal replacement therapy and antibiotic stewardship are processes that are amenable to intermittent ad hoc consultation.

Although this study was not designed to investigate health disparities, we identified important signals regarding the effect of sex and health insurance type on ICU outcome. Females with primary cardiac diagnoses were less likely than males to be admitted to the CICU. Furthermore, female sex remained an independent predictor of in-hospital mortality in both multivariate models. Similar findings have previously been identified in cohorts of general ICU patients and patients with septic shock; however, this is controversial and has not been a consistent finding.^29–31^ The association of Medicaid and lack of insurance with increased mortality also deserves mentioning. These results support previous studies demonstrating worse outcomes for Medicaid and uninsured patients in the general ICU setting and across cardiovascular diseases such as CAD and heart failure.^32–35^ These patients have previously been found to receive less aggressive care (including necessary procedures) and suffer increased mortality, perhaps due to systemic lack of health care access or inherent biases in the medical system. Further investigation into these outcome differences is warranted.

### STUDY LIMITATIONS.

The present study has inherent limitations. Due to limited variability in the included CICUs (predominantly level I centers), the findings may not be generalizable to nonteaching and community hospitals. Another significant limitation is our inability to identify the CICU staffing models of the 14 included hospitals, which were not available in the database. However, 12 of the 14 sites were designated as having level I CICUs and were thus likely to be academic centers. A prior study covering 2015 to 2016 reported that among 612 sites, 74.2% of all hospitals and 62.6% of academic centers had traditionally staffed CICUs.^22^ Thus, we would expect the hospitals we studied from 2009 to 2014 to have a similar if not larger degree of traditional staffing. Cardiovascular quality metrics (eg, door to balloon time), cardiovascular care timeliness, cardiovascular team implementation, care bundle usage, nurse-patient ratios, and nighttime coverage models were also not available in the database. Thus, these potentially important variables could not be accounted for in multivariable modeling. Although this limits our ability to unequivocally identify specific reasons for the outcome differences in CICUs and non-CICUs, the results still point to significant shortcomings of what were likely traditionally staffed CICUs (eg, without critical care medicine expertise and with multiple admitting providers).

It is also important to recognize that coded data can be misleading, as hospitals and providers may variably code diagnoses and procedures, leading to inconsistencies. For example, although it was our intent to limit analysis to patients with primary cardiac diagnoses, this could only be identified as the first listed diagnosis for a patient encounter, and thus, while appropriate, in some cases, there could exist a degree of uncertainty as to the “main” reason for hospital admission. In addition, patients requiring mechanical ventilation and vasopressors for pure cardiac conditions vs noncardiac conditions and mixed conditions represent populations with potentially disparate risk sets. As such, the findings in our subgroup analyses of patients requiring mechanical ventilation or vasopressors need to be confirmed in larger cohorts, allowing for greater risk adjustment. Finally, although our study population did not include more recent years, CICU staffing models have not substantially changed in the United States since our data were collected, and data supporting the impact of noncardiac illnesses on CICU patient outcomes continue to accumulate.

## CONCLUSIONS

In this historical cohort study, patients with a diverse range of primary cardiac diagnoses appear to have similar outcomes when admitted to a CICU as compared to a noncardiac ICU; however, the most critically ill patients with comorbid noncardiac ICU-level illnesses may fare better in a noncardiac ICU setting. The implied benefit of cardiac-specific ICU care may be reversed by increasing patient complexity and lack of access to general critical care expertise. Therefore, the continued development and standardization of CICU staffing models that incorporate critical care medicine (either with critical care-trained cardiologists or critical care medicine comanagement teams) may improve care delivery, particularly in level I CICUs.

## Supplementary Material

MMC1

## Figures and Tables

**CENTRAL ILLUSTRATION:**
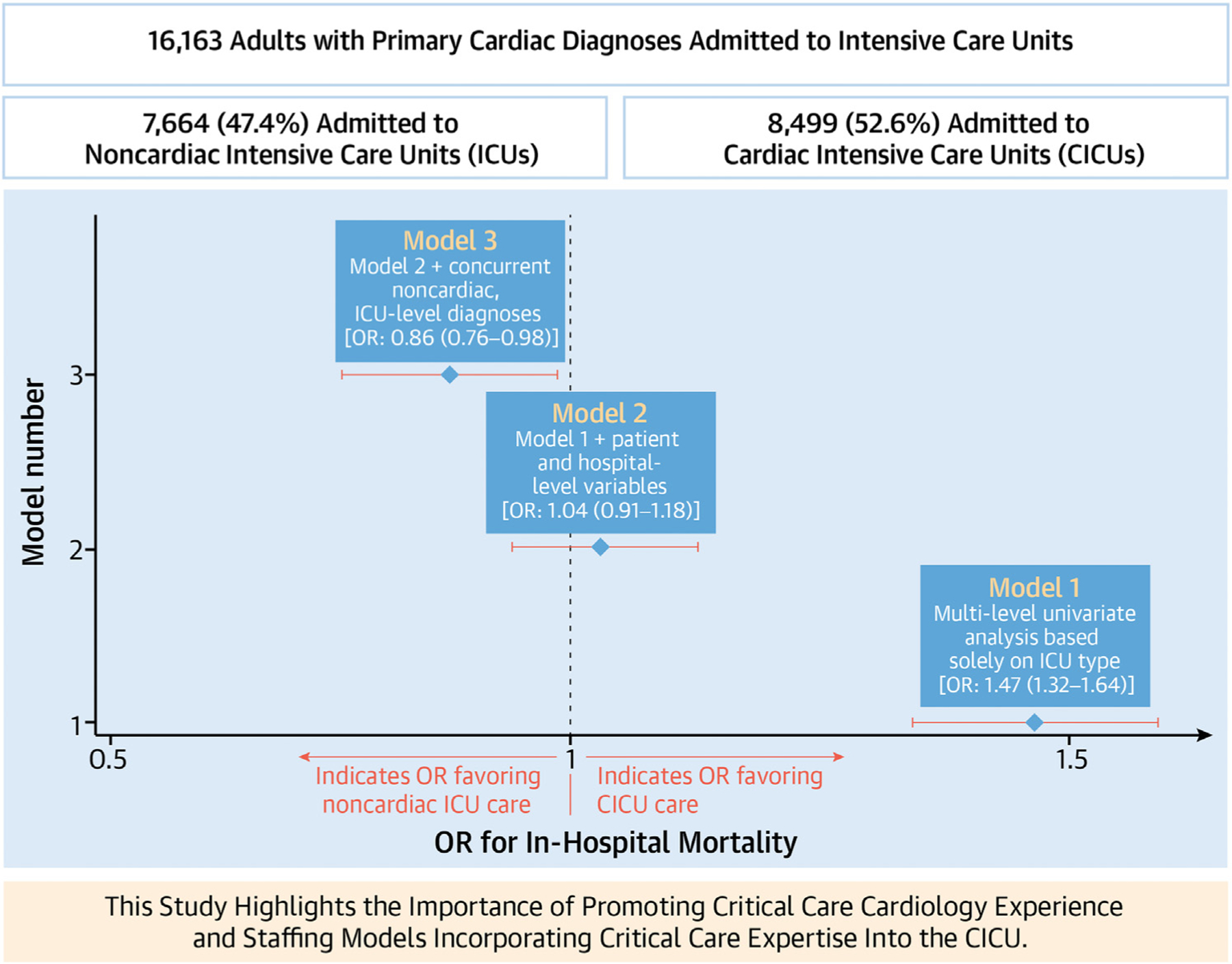
Outcomes for Cardiac Patients With Concurrent, Noncardiac Critical Illness May Improve With the Expertise Available in Noncardiac ICUs Displayed are the odds ratios (OR) and 95% CIs for inpatient mortality comparing admission to CICUs vs noncardiac ICUs, calculated using 3 different models. Univariate analysis (model 1) identified increased mortality in the noncardiac ICU group compared to the CICU group (OR: 1.47, 95% CI: 1.32–1.64; *P* < 0.0001). Multivariable analysis, incorporating patient- and hospital-level variables (model 2, covariates in Table 3), dissipated the previously identified association between ICU type and mortality (OR: 1.04, 95% CI: 0.91–1.18; *P* = 0.56). The inclusion of concurrent, noncardiac ICU-level diagnoses such as respiratory failure (model 3, covariates in Supplemental Table 4) lead to a significant reversal in the mortality trend, with improved outcomes in the noncardiac ICU group (OR: 0.86, 95% CI: 0.76–0.98; *P* = 0.03). CICU = cardiac intensive care unit..

**TABLE 1:** Patient Characteristics

	Admitted to CICU (n = 8,499)	Admitted to Non-CICU (n = 7,664)
Demographics		
Age, y	64.5 ± 13.7	66.1 ± 13.9
Female	36.8 (3,130)	39.4 (3,020)
Race		
Caucasian	78.2 (6,650)	76.7 (5,879)
African American	12.6 (1,072)	10.8 (829)
Other/specified	1.6 (132)	1.6 (125)
Unknown/unspecified	7.6 (645)	10.8 (831)
Insurance		
Medicare	42.5 (3,615)	48.6 (3,722)
Medicaid	4.4 (378)	4.8 (366)
Other insurance	21.7 (1,843)	16.8 (1,291)
Self-insured	3.7 (311)	2.2 (172)
Unknown	27.7 (2,352)	27.6 (2,113)
Clinical characteristics		
Primary cardiac diagnosis		
Acute coronary syndrome/CAD	56.9 (4,834)	31.6 (2,425)
Heart failure/shock	7.2 (608)	9.9 (762)
Cardiac arrest/arrhythmia	13.9 (1,184)	11.8 (904)
Valvular disease	3.0 (254)	8.9 (683)
Aortic/peripheral arterial disease	3.8 (322)	14.1 (1,079)
Periprocedural	1.8 (154)	3.7 (284)
No acute CV disease	0.9 (72)	4.2 (319)
Other cardiac disease	2.9 (245)	7.5 (576)
Multiple cardiac diagnoses	9.7 (826)	8.3 (632)
Elixhauser score	2.8 ± 2.4	3.2 ± 2.5
SOFA score	2.4 ± 3.1	3.7 ± 3.5
SOFA organ scores		
SOFA cardiovascular	0.7 ± 1.1	1.1 ± 1.4
SOFA respiratory	0.3 ± 0.8	0.7 ± 1.1
SOFA renal	0.6 ±0.9	0.6 ± 1.0
SOFA hepatic	0.2 ± 0.4	0.2 ± 0.5
SOFA coagulation	0.2 ± 0.5	0.4 ± 0.7
SOFA neurological	0.4 ± 1.1	0.7 ± 1.2
Intubated^a^	3.6 (303)	6.7 (514)
Vasopressors^a^	17.5 (1,486)	29.6 (2,265)
Noncardiac ICU diagnoses^b^		
Respiratory	36.1 (3,065)	58.5 (4,486)
Infectious disease/sepsis	11.6 (988)	20.5 (1,572)
Renal/metabolic/toxic	89.1 (7,575)	91.1 (6,984)
Digestive system	28.6 (2,428)	35.8 (2,740)
Skin/soft tissue/musculoskeletal	22.0 (1,873)	29.1 (2,230)
Neurologic	55.5 (4,717)	57.9 (4,434)
Trauma/procedural complication	10.1 (860)	14.5 (1,108)
Hematologic/oncologic	34.8 (2,961)	46.6 (3,569)
Hospital length of stay, d	7.4 ± 6.9	9.4 ± 9.9
ICU length of stay, d	4.0 ± 3.5	4.3 ± 4.8

Values are mean ± SD or % (n).

a Indicates that the patient was started on vasopressors or intubated within 48 h of presentation.

b Indicates the presence of a diagnosis generally necessitating ICU-level care.

CAD = coronary artery disease; CICU = cardiac intensive care unit; CV = cardiovascular; ICU = intensive care unit; SD = standard deviation; SOFA = sequential organ failure assessment.

**TABLE 2:** Hospital Characteristics

Region	
Northeast	28.6 (4)
South	28.6 (4)
Midwest	35.7 (5)
West	7.1 (1)
Local development	
Rural	28.6 (4)
Urban	71.4 (10)
Capacity (beds)	
<200	7.1 (1)
200–499	64.3 (9)
≥500	28.6 (4)
ICU level^a^	
I	85.7 (12)
II	14.3 (2)

Values are % (n). Level II centers had cardiac catheterization labs and available cardiovascular surgery but no transplant/ventricular assist device capability. Level III centers were without both cardiac catheterization labs and cardiovascular surgery programs.

a Level I hospitals were defined as those performing ECMO, and/or ventricular assist device placement, and/or heart transplant.

ECMO = extracorporeal membrane oxygenation; ICU = intensive care unit.

**TABLE 3:** OR Estimates of Inpatient Mortality for Model 2

Variable Comparisons or Unit Increase	OR	95% CI	*P* Value
Admission to non-CICU vs CICU	1.038	0.914–1.179	0.5628
Unit increase in age (y)^a^	1.031	1.026–1.036	<0.0001
Unit increase in SOFA score^a^	1.317	1.296–1.339	<0.0001
Unit increase in packs-per-day tobacco (packs)^a^	0.941	0.796–1.113	0.4774
Unit increase in Elixhauser score^a^	1.086	1.059–1.115	<0.0001
Male vs female	0.792	0.706–0.889	<0.0001
Insurance status			0.0012
Medicaid vs Medicare	1.584	1.189–2.111	
Other insurance vs Medicare	1.060	0.871–1.289	
Self-insured vs Medicare	1.683	1.145–2.472	
Unknown vs Medicare	1.307	1.047–1.632	
Race			0.0954
Caucasian vs African American	0.937	0.781–1.124	
Other/specified vs African American	1.558	1.017–2.385	
Unknown/unspecified vs African American	0.978	0.764–1.251	
Admit year			0.0655
2010 vs 2009	1.029	0.802–1.319	
2011 vs 2009	0.846	0.659–1.086	
2012 vs 2009	0.794	0.619–1.018	
2013 vs 2009	0.780	0.612–0.994	
2014 vs 2009	0.937	0.711–1.235	
Cardiac diagnosis category			<0.0001
ACS/CAD vs “other” cardiac diagnoses	0.383	0.305–0.481	
Aortic and PAD vs “other” cardiac diagnoses	0.331	0.247–0.444	
Cardiac arrest/arrhythmia vs “other” cardiac diagnoses	0.997	0.791–1.258	
Heart failure/shock vs “other” cardiac diagnoses	0.809	0.631–1.038	
Multiple cardiac diagnoses vs “other” cardiac diagnoses	0.620	0.475–0.808	
No acute cardiac diagnosis vs “other” cardiac diagnoses	0.548	0.358–0.839	
Periprocedural monitoring vs “other” cardiac diagnoses	0.435	0.288–0.657	
Valvular heart disease vs “other” cardiac diagnoses	0.159	0.113–0.224	
CICU level, I vs II	1.341	0.494–3.641	0.5653
Hospital bed size, 200–499 vs ≥500	1.653	0.691–3.952	0.2584
Hospital setting, urban vs rural	0.310	0.132–0.726	0.0070
Teaching hospital, yes vs no	0.958	0.356–2.574	0.9315
Annual admissions			0.1946
10,001–20,000 vs ≤10,000	1.386	0.599–3.211	
>20,000 vs ≤10,000	0.625	0.231–1.690	

*P* values displayed are for type III tests of fixed effects for each variable category.

a Effects of continuous variables are assessed as one unit change from the mean.

ACS = acute coronary syndrome; CAD = coronary artery disease; CICU = cardiac intensive care unit; OR = odds ratio; PAD = peripheral arterial disease; SOFA = sequential organ failure assessment.

## PERSPECTIVES

### COMPETENCY IN SYSTEMS-BASED PRACTICE AND PATIENT CARE:

CICU staffing models that prioritize critical care medicine expertise potentially offer more comprehensive care that impacts patient outcomes, especially in the setting of multiorgan failure and concurrent noncardiac illnesses.

### TRANSLATIONAL OUTLOOK:

Future studies should investigate the implications of transitioning from traditionally staffed CICUs to CICUs with critical care medicine expertise and further explore how critical care medicine expertise is best incorporated.

## ABBREVIATIONS AND ACRONYMS

ACS: acute coronary syndrome
CAD: coronary artery disease
CCU: coronary care unit
CICU: cardiac intensive care unit
ICD-9: International Classification of Diseases-9th Revision
OR: odds ratio
SOFA: Sequential Organ Failure Assessment

## References

[1] Brown KW, Macmillan RL, Forbath N, Melgrano F, Scott JW. Coronary unit: an intensive-care centre for acute myocardial infarction. Lancet 1963;2:349–352. 14044279 10.1016/s0140-6736(63)93011-3

[2] Killip T 3rd, Kimball JT. Treatment of myocardial infarction in a coronary care unit. A two year experience with 250 patients. Am J Cardiol 1967;20:457–464. 6059183 10.1016/0002-9149(67)90023-9

[3] Rotstein Z, Mandelzweig L, Lavi B, Eldar M, Gottlieb S, Hod H. Does the coronary care unit improve prognosis of patients with acute myocardial infarction? A thrombolytic era study. Eur Heart J 1999;20:813–818. 10329079 10.1053/euhj.1998.1452

[4] Holland EM, Moss TJ. Acute noncardiovascular illness in the cardiac intensive care unit. J Am Coll Cardiol 2017;69:1999–2007. 28427574 10.1016/j.jacc.2017.02.033

[5] Morrow DA, Fang JC, Fintel DJ, Evolution of critical care cardiology: transformation of the cardiovascular intensive care unit and the emerging need for new medical staffing and training models: a scientific statement from the American Heart Association. Circulation 2012;126: 1408–1428. 22893607 10.1161/CIR.0b013e31826890b0

[6] Katz JN, Shah BR, Volz EM, Evolution of the coronary care unit: clinical characteristics and temporal trends in healthcare delivery and outcomes. Crit Care Med 2010;38:375–381. 20029344 10.1097/CCM.0b013e3181cb0a63

[7] Sinha SS, Sjoding MW, Sukul D, Changes in primary noncardiac diagnoses over time among elderly cardiac intensive care unit patients in the United States. Circ Cardiovasc Qual Outcomes 2017;10:e003616. 28794121 10.1161/CIRCOUTCOMES.117.003616 PMC5657300

[8] Bohula EA, Katz JN, van Diepen S, Demographics, care patterns, and outcomes of patients admitted to cardiac intensive care units: the critical care cardiology trials network prospective North American multicenter registry of cardiac critical illness. JAMA Cardiol 2019;4(9):928–935. 31339509 10.1001/jamacardio.2019.2467 PMC6659157

[9] Berg DD, Bohula EA, van Diepen S, Epidemiology of shock in contemporary cardiac intensive care units. Circ Cardiovasc Qual Outcomes 2019;12:e005618. 30879324 10.1161/CIRCOUTCOMES.119.005618 PMC11032172

[10] Jentzer JC, Ahmed AM, Vallabhajosyula S, Shock in the cardiac intensive care unit: changes in epidemiology and prognosis over time. Am Heart J 2021;232:94–104. 33257304 10.1016/j.ahj.2020.10.054

[11] Metkus TS, Miller PE, Alviar CL, Advanced respiratory support in the contemporary cardiac ICU. Crit Care Explor 2020;2:e0182. 33235999 10.1097/CCE.0000000000000182 PMC7678799

[12] van Diepen S, Tran DT, Ezekowitz JA, The high cost of critical care unit over-utilization for patients with NSTE ACS. Am Heart J 2018;202: 84–88. 29906667 10.1016/j.ahj.2018.05.003

[13] Kapoor K, Verceles AC, Netzer G, A collaborative cardiologist-intensivist management model improves cardiac intensive care unit outcomes. J Am Coll Cardiol 2017;70:1422–1423. 28882242 10.1016/j.jacc.2017.07.739

[14] Na SJ, Chung CR, Jeon K, Association between presence of a cardiac intensivist and mortality in an adult cardiac care unit. J Am Coll Cardiol 2016;68:2637–2648. 27978948 10.1016/j.jacc.2016.09.947

[15] Brusca SB, Barnett C, Barnhart BJ, Role of critical care medicine training in the cardiovascular intensive care unit: survey responses from dual certified critical care cardiologists. J Am Heart Assoc 2019;8:e011721. 30879373 10.1161/JAHA.118.011721 PMC6475069

[16] Berg DD, Barnett CF, Kenigsberg BB, Clinical practice patterns in temporary mechanical circulatory support for shock in the critical care cardiology trials network (CCCTN) registry. Circ Heart Fail 2019;12:e006635. 31707801 10.1161/CIRCHEARTFAILURE.119.006635 PMC7008928

[17] Miller PE, Chouairi F, Thomas A, Transition from an open to closed staffing model in the cardiac intensive care unit improves clinical outcomes. J Am Heart Assoc 2021;10:e018182. 33412899 10.1161/JAHA.120.018182 PMC7955420

[18] Miller PE, Thomas A, Breen TJ, Prevalence of noncardiac multimorbidity in patients admitted to two cardiac intensive care units and their association with mortality. Am J Med 2021;134:653–661.e5. 33129785 10.1016/j.amjmed.2020.09.035 PMC8079541

[19] Nandiwada S, Islam S, Jentzer JC, The association between cardiac intensive care unit mechanical ventilation volumes and in-hospital mortality. Eur Heart J Acute Cardiovasc Care 2021;10:797–805. 34318875 10.1093/ehjacc/zuab055 PMC9067446

[20] Jentzer JC, Lawler PR, van Diepen S, Systemic inflammatory response syndrome is associated with increased mortality across the spectrum of shock severity in cardiac intensive care patients. Circ Cardiovasc Qual Outcomes 2020;13:e006956. 33280435 10.1161/CIRCOUTCOMES.120.006956

[21] Padkins M, Breen T, Van Diepen S, Barsness G, Kashani K, Jentzer JC. Incidence and outcomes of acute kidney injury stratified by cardiogenic shock severity. Catheter Cardiovasc Interv 2021;98:330–340. 33825337 10.1002/ccd.29692

[22] van Diepen S, Fordyce CB, Wegermann ZK, Organizational structure, staffing, resources, and educational initiatives in cardiac intensive care units in the United States: an American Heart Association Acute Cardiac Care Committee and American College of Cardiology Critical Care Cardiology Working Group cross-sectional survey. Circ Cardiovasc Qual Outcomes 2017;10:e003864. 28794122 10.1161/CIRCOUTCOMES.117.003864 PMC5666693

[23] Lagu T, Pekow PS, Shieh MS, Validation and comparison of seven mortality prediction models for hospitalized patients with acute decompensated heart failure. Circ Heart Fail 2016;9:e002912. 10.1161/CIRCHEARTFAILURE.115.002912 27514749 PMC4988343

[24] Pepper DJ, Demirkale CY, Sun J, Does obesity protect against death in sepsis? A retrospective cohort study of 55,038 adult patients. Crit Care Med 2019;47:643–650. 30789403 10.1097/CCM.0000000000003692 PMC6465121

[25] Camp BW. 2012 ICD-9-CM Volume 1 (Numeric Index of Diseases and Injuries) (Know The Code Book 2) BillingCoodingLibrary.com; 2012.

[26] Thompson NR, Fan Y, Dalton JE, A new Elixhauser-based comorbidity summary measure to predict in-hospital mortality. Med Care 2015;53:374–379. 25769057 10.1097/MLR.0000000000000326 PMC4812819

[27] Saeed M, Villarroel M, Reisner AT, Multiparameter intelligent monitoring in intensive care II: a public-access intensive care unit database. Crit Care Med 2011;39:952–960. 21283005 10.1097/CCM.0b013e31820a92c6 PMC3124312

[28] The Fuster V. (R)evolution of the CICU: better for the patient, better for education. J Am Coll Cardiol 2018;72:2269–2271. 30360831 10.1016/j.jacc.2018.09.018

[29] Hollinger A, Gayat E, Feliot E, Gender and survival of critically ill patients: results from the FROG-ICU study. Ann Intensive Care 2019;9:43. 30927096 10.1186/s13613-019-0514-y PMC6441070

[30] Mahmood K, Eldeirawi K, Wahidi MM. Association of gender with outcomes in critically ill patients. Crit Care 2012;16:R92. 22617003 10.1186/CC11355 PMC3580638

[31] Pietropaoli AP, Glance LG, Oakes D, Fisher SG. Gender differences in mortality in patients with severe sepsis or septic shock. Gend Med 2010;7: 422–437. 21056869 10.1016/j.genm.2010.09.005 PMC3322379

[32] Fowler-Brown A, Corbie-Smith G, Garrett J, Lurie N. Risk of cardiovascular events and death–does insurance matter? J Gen Intern Med 2007;22:502–507. 17372800 10.1007/s11606-007-0127-2 PMC1829431

[33] Kapoor JR, Kapoor R, Hellkamp AS, Hernandez AF, Heidenreich PA, Fonarow GC. Payment source, quality of care, and outcomes in patients hospitalized with heart failure. J Am Coll Cardiol 2011;58:1465–1471. 21939830 10.1016/j.jacc.2011.06.034 PMC4603423

[34] Lyon SM, Benson NM, Cooke CR, Iwashyna TJ, Ratcliffe SJ, Kahn JM. The effect of insurance status on mortality and procedural use in critically ill patients. Am J Respir Crit Care Med 2011;184: 809–815. 21700910 10.1164/rccm.201101-0089OC PMC3208649

[35] Smolderen KG, Spertus JA, Tang F, Treatment differences by health insurance among outpatients with coronary artery disease: insights from the national cardiovascular data registry. J Am Coll Cardiol 2013;61:1069–1075. 23375933 10.1016/j.jacc.2012.11.058 PMC3641586

