# Supplementary material for: Outcomes of Patients With Primary Cardiac Diagnoses Admitted to Cardiac vs Noncardiac Intensive Care Units
Source: JACC Adv. Author manuscript; Available in PMC 2022 Dec 2. (PMC9718493; doi:10.1016/j.jacadv.2022.100114)
Supplement: MMC1 [file NIHMS1841831-supplement-MMC1.pdf]

Supplemental Table 1: Initial List of Diagnosis Codes Used for Data Cleaning

Number	ICD-9 Code	Diagnosis Description
1	194.6	Malignant Neoplasm of Aortic Body and Other Paraganglia
2	249.21	Secondary Diabetes Mellitus with Hyperosmolarity, Uncontrolled
3	250	Diabetes Mellitus
4	250.00	Diabetes mellitus without mention of complication, type II or unspecified type, not stated as uncontrolled
5	250.01	Diabetes Mellitus without Mention of Complication, Type I [juvenile Type], Not Stated as Uncontrolled
6	250.02	Diabetes mellitus without mention of complication, type II or unspecified type, uncontrolled
7	250.03	Diabetes Mellitus without Mention of Complication, Type I [juvenile Type], Uncontrolled
8	250.1	Diabetes with Ketoacidosis
9	250.10	Diabetes Mellitus with Ketoacidosis, Type II or Unspecified Type, Not Stated as Uncontrolled
10	250.11	Diabetes Mellitus with Ketoacidosis, Type I [juvenile Type], Not Stated as Uncontrolled
11	250.12	Diabetes mellitus with ketoacidosis, type II or unspecified type, uncontrolled
12	250.13	Diabetes Mellitus with Ketoacidosis, Type I [juvenile Type], Uncontrolled
13	250.2	Diabetes Mellitus with Hyperosmolarity
14	250.20	Diabetes mellitus with hyperosmolarity, type II or unspecified type, not stated as uncontrolled
15	250.21	Diabetes Mellitus with Hyperosmolarity, Type I [juvenile Type], Not Stated as Uncontrolled
16	250.22	Diabetes mellitus with hyperosmolarity, type II or unspecified type, uncontrolled
17	250.23	Diabetes Mellitus with Hyperosmolarity, Type I [juvenile Type], Uncontrolled
18	250.30	Diabetes mellitus with other coma, type II or unspecified type, not stated as uncontrolled
19	250.31	Diabetes Mellitus with Other Coma, Type I [juvenile Type], Not Stated as Uncontrolled
20	250.32	Diabetes mellitus with other coma, type II or unspecified type, uncontrolled
21	250.33	Diabetes Mellitus with Other Coma, Type I [juvenile Type], Uncontrolled
22	250.40	Diabetes mellitus with renal manifestations, type II or unspecified type, not stated as uncontrolled
23	250.41	Diabetes Mellitus with Renal Manifestations, Type I [juvenile Type], Not Stated as Uncontrolled
24	250.42	Diabetes mellitus with renal manifestations, type II or unspecified type, uncontrolled
25	250.50	Diabetes mellitus with ophthalmic manifestations, type II or unspecified type, not stated as uncontrolled
26	250.61	Diabetes Mellitus with Neurological Manifestations, Type I [juvenile Type], Not Stated as Uncontrolled
27	250.63	Diabetes Mellitus with Neurological Manifestations, Type I [juvenile Type], Uncontrolled
28	250.70	Diabetes Mellitus with Peripheral Circulatory Disorders, Type II or Unspecified Type, Not Stated as Uncontrolled
29	250.72	Diabetes mellitus with peripheral circulatory disorders, type II or unspecified type, uncontrolled
30	250.73	Diabetes Mellitus with Peripheral Circulatory Disorders, Type I [juvenile Type], Uncontrolled
31	250.80	Diabetes mellitus with other specified manifestations, type II or unspecified type, not stated as uncontrolled
32	250.81	Diabetes Mellitus with Other Specified Manifestations, Type I [juvenile Type], Not Stated as Uncontrolled
33	250.82	Diabetes mellitus with other specified manifestations, type II or unspecified type, uncontrolled
34	250.83	Diabetes Mellitus with Other Specified Manifestations, Type I [juvenile Type], Uncontrolled
35	250.90	Diabetes mellitus with unspecified complication, type II or unspecified type, not stated as uncontrolled
36	250.91	Diabetes Mellitus with Unspecified Complication, Type I [juvenile Type], Not Stated as Uncontrolled
37	250.92	Diabetes mellitus with unspecified complication, type II or unspecified type, uncontrolled
38	276.50	Volume Depletion, Unspecified
39	276.52	Hypovolemia
40	285.1	Acute Posthemorrhagic Anemia
41	303	Alcohol Dependence Syndrome
42	303.00	Acute Alcoholic Intoxication in Alcoholism, Unspecified Drinking Behavior

43	305.00	Alcohol Abuse, Unspecified Drinking Behavior
44	305.1	Tobacco Use Disorder
45	337.29	Reflex Sympathetic Dystrophy of Other Specified Site
46	362.11	Hypertensive Retinopathy
47	401	Essential Hypertension
48	401.1	Benign Essential Hypertension
49	401.9	Unspecified Essential Hypertension
50	403.10	Hypertensive Chronic Kidney Disease, Benign, with Chronic Kidney Disease Stage I Through Stage IV, or Unspecified
51	403.11	Hypertensive Chronic Kidney Disease, Benign, with Chronic Kidney Disease Stage V or End Stage Renal Disease
52	405.91	Unspecified Renovascular Hypertension
53	405.99	Other Unspecified Secondary Hypertension
54	415.1	Pulmonary Embolism and Infarction
55	415.11	Iatrogenic Pulmonary Embolism and Infarction
56	415.13	Saddle Embolus of Pulmonary Artery
57	415.19	Other Pulmonary Embolism and Infarction
58	416.2	Chronic Pulmonary Embolism
59	417.8	Other Specified Diseases of Pulmonary Circulation
60	430	Subarachnoid Hemorrhage
61	431	Intracerebral Hemorrhage
62	432.0	Nontraumatic Extradural Hemorrhage
63	432.9	Unspecified Intracranial Hemorrhage
64	433.00	Occlusion and Stenosis of Basilar Artery, without Mention of Cerebral Infarction
65	433.01	Occlusion and Stenosis of Basilar Artery, with Cerebral Infarction
66	433.10	Occlusion and Stenosis of Carotid Artery, without Mention of Cerebral Infarction
67	433.11	Occlusion and Stenosis of Carotid Artery, with Cerebral Infarction
68	433.20	Occlusion and Stenosis of Vertebral Artery, without Mention of Cerebral Infarction
69	433.21	Occlusion and Stenosis of Vertebral Artery, with Cerebral Infarction
70	433.30	Occlusion and Stenosis of Multiple and Bilateral Precerebral Arteries, without Mention of Cerebral Infarction
71	433.31	Occlusion and Stenosis of Multiple and Bilateral Precerebral Arteries, with Cerebral Infarction
72	433.80	Occlusion and Stenosis of Other Specified Precerebral Artery, without Mention of Cerebral Infarction
73	433.81	Occlusion and Stenosis of Other Specified Precerebral Artery, with Cerebral Infarction
74	434.00	Cerebral Thrombosis, without Mention of Cerebral Infarction
75	434.01	Cerebral Thrombosis, with Cerebral Infarction
76	434.10	Cerebral Embolism, without Mention of Cerebral Infarction
77	434.11	Cerebral Embolism, with Cerebral Infarction
78	434.90	Cerebral Artery Occlusion, Unspecified, without Mention of Cerebral Infarction
79	434.91	Cerebral Artery Occlusion, Unspecified, with Cerebral Infarction
80	435.8	Other Specified Transient Cerebral Ischemias
81	435.9	Unspecified Transient Cerebral Ischemia
82	436	Acute, but Ill-Defined, Cerebrovascular Disease
83	437.0	Cerebral Atherosclerosis
84	437.2	Hypertensive Encephalopathy
85	437.8	Other Ill-Defined Cerebrovascular Disease
86	437.9	Unspecified Cerebrovascular Disease
87	438.9	Unspecified Late Effects of Cerebrovascular Disease

88	453.40	Acute Venous Embolism and Thrombosis of Unspecified Deep Vessels of Lower Extremity
89	453.41	Acute Venous Embolism and Thrombosis of Deep Vessels of Proximal Lower Extremity
90	453.51	Chronic Venous Embolism and Thrombosis of Deep Vessels of Proximal Lower Extremity
91	453.77	Chronic Venous Embolism and Thrombosis of Other Thoracic Veins
92	453.9	Embolism and Thrombosis of Unspecified Site
93	557.9	Unspecified Vascular Insufficiency of Intestine
94	642.01	Benign Essential Hypertension Complicating Pregnancy, Childbirth, and the Puerperium, Delivered, with or without Mention of Antepartum Condition
95	642.03	Benign Essential Hypertension Complicating Pregnancy, Childbirth, and the Puerperium, Antepartum Condition or Complication
96	642.04	Benign Essential Hypertension Complicating Pregnancy, Childbirth, and the Puerperium, Postpartum Condition or Complication
97	642.13	Hypertension Secondary to Renal Disease, Complicating Pregnancy, Childbirth, and the Puerperium, Antepartum Condition or Complication
98	642.14	Hypertension Secondary to Renal Disease, Complicating Pregnancy, Childbirth, and the Puerperium, Postpartum Condition or Complication
99	642.21	Other Pre-Existing Hypertension Complicating Pregnancy, Childbirth, and the Puerperium, Delivered, with or without Mention of Antepartum Condition
100	642.23	Other Pre-Existing Hypertension Complicating Pregnancy, Childbirth, and the Puerperium, Antepartum Condition or Complication
101	642.24	Other Pre-Existing Hypertension Complicating Pregnancy, Childbirth, and the Puerperium, Postpartum Condition or Complication
102	642.31	Transient Hypertension of Pregnancy, Delivered, with or without Mention of Antepartum Condition
103	642.73	Pre-Eclampsia or Eclampsia Superimposed on Pre-Existing Hypertension, Antepartum Condition or Complication
104	642.74	Pre-Eclampsia or Eclampsia Superimposed on Pre-Existing Hypertension, Postpartum Condition or Complication
105	642.91	Unspecified Hypertension Complicating Pregnancy, Childbirth, or the Puerperium, Delivered, with or without Mention of Antepartum Condition
106	642.93	Unspecified Hypertension Complicating Pregnancy, Childbirth, or the Puerperium, Antepartum Condition or Complication
107	642.94	Unspecified Hypertension Complicating Pregnancy, Childbirth, or the Puerperium, Postpartum Condition or Complication
108	648.03	Diabetes Mellitus Complicating Pregnancy, Childbirth, or the Puerperium, Antepartum Condition or Complication
109	796.2	Elevated Blood Pressure Reading without Diagnosis of Hypertension
110	861.02	Laceration of Heart without Penetration of Heart Chambers or Open Wound Into Thorax
111	861.10	Unspecified Injury of Heart with Open Wound Into Thorax
112	861.12	Laceration of Heart without Penetration of Heart Chambers, with Open Wound Into Thorax
113	861.13	Laceration of Heart with Penetration of Heart Chambers and Open Wound Into Thorax
114	901.0	Injury to Thoracic Aorta
115	901.40	Injury to Pulmonary Vessel(s), Unspecified
116	901.81	Injury to Intercostal Artery or Vein
117	965.01	Poisoning by Heroin
118	965.09	Poisoning by Other Opiates and Related Narcotics
119	965.4	Poisoning by Aromatic Analgesics, Not Elsewhere Classified
120	969.4	Poisoning by Benzodiazepine-Based Tranquilizers
121	996.82	Complications of Transplanted Liver
122	996.84	Complications of Transplanted Lung
123	996.85	Complications of Bone Marrow Transplant
124	996.86	Complications of Transplanted Pancreas
125	996.89	Complications of Other Transplanted Organ
126	997.91	Hypertension
127	V11.3	Personal History of Alcoholism
128	V15.82	Personal History of Tobacco Use
129	V42.7	Liver Replaced by Transplant
130	V42.83	Pancreas Replaced by Transplant
131	074.21	Coxsackie Pericarditis
132	112.81	Candidal Endocarditis

133	164.1	Malignant Neoplasm of Heart
134	212.7	Benign Neoplasm of Heart
135	272.0	Pure Hypercholesterolemia
136	272.2	Mixed Hyperlipidemia
137	272.4	Other and Unspecified Hyperlipidemia
138	272.6	Lipodystrophy
139	272.7	Lipidoses
140	272.9	Unspecified Disorder of Lipoid Metabolism
141	276.6	Fluid Overload
142	337.01	Carotid Sinus Syndrome
143	391.0	Acute Rheumatic Pericarditis
144	391.1	Acute Rheumatic Endocarditis
145	391.8	Other Acute Rheumatic Heart Disease
146	394	Diseases of Mitral Valve
147	394.0	Mitral Stenosis
148	394.1	Rheumatic Mitral Insufficiency
149	394.2	Mitral Stenosis with Insufficiency
150	394.9	Other and Unspecified Mitral Valve Diseases
151	395.0	Rheumatic Aortic Stenosis
152	395.1	Rheumatic Aortic Insufficiency
153	395.2	Rheumatic Aortic Stenosis with Insufficiency
154	396.0	Mitral Valve Stenosis and Aortic Valve Stenosis
155	396.1	Mitral Valve Stenosis and Aortic Valve Insufficiency
156	396.2	Mitral Valve Insufficiency and Aortic Valve Stenosis
157	396.3	Mitral Valve Insufficiency and Aortic Valve Insufficiency
158	396.8	Multiple Involvement of Mitral and Aortic Valves
159	396.9	Mitral and Aortic Valve Diseases, Unspecified
160	397.0	Diseases of Tricuspid Valve
161	398.90	Rheumatic Heart Disease, Unspecified
162	398.91	Rheumatic Heart Failure (Congestive)
163	401.0	Malignant Essential Hypertension
164	402.00	Hypertensive Heart Disease, Malignant, without Heart Failure
165	402.01	Hypertensive Heart Disease, Malignant, with Heart Failure
166	402.10	Hypertensive Heart Disease, Benign, without Heart Failure
167	402.11	Hypertensive Heart Disease, Benign, with Heart Failure
168	402.90	Hypertensive Heart Disease, Unspecified, without Heart Failure
169	402.91	Hypertensive Heart Disease, Unspecified, with Heart Failure
170	404.00	Hypertensive Heart and Chronic Kidney Disease, Malignant, without Heart Failure and with Chronic Kidney Disease Stage I Through Stage IV, or Unspecified
171	404.02	Hypertensive Heart and Chronic Kidney Disease, Malignant, without Heart Failure and with Chronic Kidney Disease Stage V or End Stage Renal Disease
172	404.11	Hypertensive Heart and Chronic Kidney Disease, Benign, with Heart Failure and with Chronic Kidney Disease Stage I Through Stage IV, or Unspecified
173	404.12	Hypertensive Heart and Chronic Kidney Disease, Benign, without Heart Failure and with Chronic Kidney Disease Stage V or End Stage Renal Disease
174	404.13	Hypertensive Heart and Chronic Kidney Disease, Benign, with Heart Failure and Chronic Kidney Disease Stage V or End Stage Renal Disease
175	404.90	Hypertensive Heart and Chronic Kidney Disease, Unspecified, without Heart Failure and with Chronic Kidney Disease Stage I Through Stage IV, or Unspecified
176	404.92	Hypertensive Heart and Chronic Kidney Disease, Unspecified, without Heart Failure and with Chronic Kidney Disease Stage V or End Stage Renal Disease
177	405.01	Malignant Renovascular Hypertension

178	410	Acute Myocardial Infarction
179	410.0	Acute Myocardial Infarction, of Anterolateral Wall
180	410.00	Acute Myocardial Infarction, of Anterolateral Wall, Episode of Care Unspecified
181	410.01	Acute Myocardial Infarction, of Anterolateral Wall, Initial Episode of Care
182	410.02	Acute Myocardial Infarction, of Anterolateral Wall, Subsequent Episode of Care
183	410.10	Acute Myocardial Infarction, of Other Anterior Wall, Episode of Care Unspecified
184	410.11	Acute Myocardial Infarction, of Other Anterior Wall, Initial Episode of Care
185	410.12	Acute Myocardial Infarction, of Other Anterior Wall, Subsequent Episode of Care
186	410.20	Acute Myocardial Infarction, of Inferolateral Wall, Episode of Care Unspecified
187	410.21	Acute Myocardial Infarction, of Inferolateral Wall, Initial Episode of Care
188	410.22	Acute Myocardial Infarction, of Inferolateral Wall, Subsequent Episode of Care
189	410.3	Acute Myocardial Infarction, of Inferoposterior Wall
190	410.30	Acute Myocardial Infarction, of Inferoposterior Wall, Episode of Care Unspecified
191	410.31	Acute Myocardial Infarction, of Inferoposterior Wall, Initial Episode of Care
192	410.32	Acute Myocardial Infarction, of Inferoposterior Wall, Subsequent Episode of Care
193	410.4	Acute Myocardial Infarction, of Other Inferior Wall
194	410.40	Acute Myocardial Infarction, of Other Inferior Wall, Episode of Care Unspecified
195	410.41	Acute Myocardial Infarction, of Other Inferior Wall, Initial Episode of Care
196	410.42	Acute Myocardial Infarction, of Other Inferior Wall, Subsequent Episode of Care
197	410.50	Acute Myocardial Infarction, of Other Lateral Wall, Episode of Care Unspecified
198	410.51	Acute Myocardial Infarction, of Other Lateral Wall, Initial Episode of Care
199	410.61	Acute Myocardial Infarction, True Posterior Wall Infarction, Initial Episode of Care
200	410.7	Acute Myocardial Infarction, Subendocardial Infarction
201	410.70	Acute Myocardial Infarction, Subendocardial Infarction, Episode of Care Unspecified
202	410.71	Acute Myocardial Infarction, Subendocardial Infarction, Initial Episode of Care
203	410.72	Acute Myocardial Infarction, Subendocardial Infarction, Subsequent Episode of Care
204	410.80	Acute Myocardial Infarction, of Other Specified Sites, Episode of Care Unspecified
205	410.81	Acute Myocardial Infarction, of Other Specified Sites, Initial Episode of Care
206	410.82	Acute Myocardial Infarction, of Other Specified Sites, Subsequent Episode of Care
207	410.9	Acute Myocardial Infarction, Unspecified Site
208	410.90	Acute Myocardial Infarction, Unspecified Site, Episode of Care Unspecified
209	410.91	Acute Myocardial Infarction, Unspecified Site, Initial Episode of Care
210	410.92	Acute Myocardial Infarction, Unspecified Site, Subsequent Episode of Care
211	411.0	Postmyocardial Infarction Syndrome
212	411.1	Intermediate Coronary Syndrome
213	411.81	Acute Coronary Occlusion without Myocardial Infarction
214	411.89	Other Acute and Subacute Forms of Ischemic Heart Disease
215	412	Old Myocardial Infarction
216	413	Angina Pectoris
217	413.0	Angina Decubitus
218	413.1	Prinzmetal Angina
219	413.9	Other and Unspecified Angina Pectoris
220	414.0	Coronary Atherosclerosis
221	414.00	Coronary Atherosclerosis of Unspecified Type of Vessel, Native or Graft
222	414.01	Coronary Atherosclerosis of Native Coronary Artery

223	414.02	Coronary Atherosclerosis of Autologous Vein Bypass Graft
224	414.04	Coronary Atherosclerosis of Artery Bypass Graft
225	414.05	Coronary Atherosclerosis of Unspecified Type of Bypass Graft
226	414.06	Coronary Atherosclerosis of Native Coronary Artery of Transplanted Heart
227	414.10	Aneurysm of Heart (Wall)
228	414.11	Aneurysm of Coronary Vessels
229	414.12	Dissection of Coronary Artery
230	414.19	Other Aneurysm of Heart
231	414.2	Chronic Total Occlusion of Coronary Artery
232	414.4	Coronary Atherosclerosis Due to Calcified Coronary Lesion
233	414.8	Other Specified Forms of Chronic Ischemic Heart Disease
234	414.9	Chronic Ischemic Heart Disease, Unspecified
235	415.0	Acute Cor Pulmonale
236	416.0	Primary Pulmonary Hypertension
237	416.9	Chronic Pulmonary Heart Disease, Unspecified
238	417.1	Aneurysm of Pulmonary Artery
239	420	Acute Pericarditis
240	420.90	Acute Pericarditis, Unspecified
241	421.0	Acute and Subacute Bacterial Endocarditis
242	421.9	Acute Endocarditis, Unspecified
243	422.90	Acute Myocarditis, Unspecified
244	422.91	Idiopathic Myocarditis
245	422.99	Other Acute Myocarditis
246	423.0	Hemopericardium
247	423.1	Adhesive Pericarditis
248	423.2	Constrictive Pericarditis
249	423.3	Cardiac Tamponade
250	423.8	Other Specified Diseases of Pericardium
251	423.9	Unspecified Disease of Pericardium
252	424.0	Mitral Valve Disorders
253	424.1	Aortic Valve Disorders
254	424.2	Tricuspid Valve Disorders, Specified as Nonrheumatic
255	424.3	Pulmonary Valve Disorders
256	424.90	Endocarditis, Valve Unspecified, Unspecified Cause
257	424.91	Endocarditis in Diseases Classified Elsewhere
258	425	Cardiomyopathy
259	425.1	Hypertrophic Cardiomyopathy
260	425.11	Hypertrophic Obstructive Cardiomyopathy
261	425.18	Other Hypertrophic Cardiomyopathy
262	425.3	Endocardial Fibroelastosis
263	425.4	Other Primary Cardiomyopathies
264	425.5	Alcoholic Cardiomyopathy
265	425.8	Cardiomyopathy in Other Diseases Classified Elsewhere
266	425.9	Secondary Cardiomyopathy, Unspecified
267	426.0	Atrioventricular Block, Complete

268	426.10	Atrioventricular Block, Unspecified
269	426.11	First Degree Atrioventricular Block
270	426.12	Mobitz (Type) II Atrioventricular Block
271	426.13	Other Second Degree Atrioventricular Block
272	426.2	Left Bundle Branch Hemiblock
273	426.3	Other Left Bundle Branch Block
274	426.4	Right Bundle Branch Block
275	426.50	Bundle Branch Block, Unspecified
276	426.51	Right Bundle Branch Block and Left Posterior Fascicular Block
277	426.52	Right Bundle Branch Block and Left Anterior Fascicular Block
278	426.53	Other Bilateral Bundle Branch Block
279	426.54	Trifascicular Block
280	426.6	Other Heart Block
281	426.7	Anomalous Atrioventricular Excitation
282	426.82	Long Qt Syndrome
283	426.89	Other Specified Conduction Disorders
284	426.9	Conduction Disorder, Unspecified
285	427.0	Paroxysmal Supraventricular Tachycardia
286	427.1	Paroxysmal Ventricular Tachycardia
287	427.2	Paroxysmal Tachycardia, Unspecified
288	427.3	Atrial Fibrillation and Flutter
289	427.31	Atrial Fibrillation
290	427.32	Atrial Flutter
291	427.41	Ventricular Fibrillation
292	427.42	Ventricular Flutter
293	427.5	Cardiac Arrest
294	427.60	Premature Beats, Unspecified
295	427.61	Supraventricular Premature Beats
296	427.69	Other Premature Beats
297	427.81	Sinoatrial Node Dysfunction
298	427.89	Other Specified Cardiac Dysrhythmias
299	427.9	Cardiac Dysrhythmia, Unspecified
300	428	Heart Failure
301	428.0	Congestive Heart Failure, Unspecified
302	428.1	Left Heart Failure
303	428.2	Systolic Heart Failure
304	428.20	Unspecified Systolic Heart Failure
305	428.21	Acute Systolic Heart Failure
306	428.22	Chronic Systolic Heart Failure
307	428.23	Acute on Chronic Systolic Heart Failure
308	428.3	Diastolic Heart Failure
309	428.30	Unspecified Diastolic Heart Failure
310	428.31	Acute Diastolic Heart Failure
311	428.32	Chronic Diastolic Heart Failure
312	428.33	Acute on Chronic Diastolic Heart Failure

313	428.40	Unspecified Combined Systolic and Diastolic Heart Failure
314	428.41	Acute Combined Systolic and Diastolic Heart Failure
315	428.42	Chronic Combined Systolic and Diastolic Heart Failure
316	428.43	Acute on Chronic Combined Systolic and Diastolic Heart Failure
317	428.9	Heart Failure, Unspecified
318	429.0	Myocarditis, Unspecified
319	429.1	Myocardial Degeneration
320	429.2	Cardiovascular Disease, Unspecified
321	429.3	Cardiomegaly
322	429.4	Functional Disturbances Following Cardiac Surgery
323	429.5	Rupture of Chordae Tendineae
324	429.6	Rupture of Papillary Muscle
325	429.71	Cardiac Septal Defect, Sequela of Myocardial Infarction
326	429.79	Other Sequelae of Myocardial Infarction, Not Elsewhere Classified
327	429.83	Takotsubo Syndrome
328	429.89	Other Ill-Defined Heart Diseases
329	429.9	Heart Disease, Unspecified
330	440	Atherosclerosis
331	440.0	Atherosclerosis of Aorta
332	440.20	Atherosclerosis of Native Arteries of the Extremities, Unspecified
333	440.21	Atherosclerosis of Native Arteries of the Extremities with Intermittent Claudication
334	440.22	Atherosclerosis of the Extremities with Rest Pain
335	440.23	Atherosclerosis of Native Arteries of the Extremities with Ulceration
336	440.24	Atherosclerosis of Native Arteries of the Extremities with Gangrene
337	440.30	Atherosclerosis of Bypass Graft of the Extremities, Unspecified Graft
338	440.31	Atherosclerosis of Autologous Vein Bypass Graft of the Extremities
339	440.32	Atherosclerosis of Nonautologous Biological Bypass Graft of the Extremities
340	440.8	Atherosclerosis of Other Specified Arteries
341	441.00	Dissecting Aneurysm of Aorta, Unspecified Site
342	441.01	Dissecting Aneurysm of Thoracic Aorta
343	441.03	Dissecting Aneurysm of Thoracoabdominal Aorta
344	441.1	Thoracic Aortic Aneurysm, Ruptured
345	441.2	Thoracic Aortic Aneurysm without Mention of Rupture
346	441.3	Abdominal Aortic Aneurysm, Ruptured
347	441.4	Abdominal Aortic Aneurysm without Mention of Rupture
348	441.5	Aortic Aneurysm of Unspecified Site, Ruptured
349	441.6	Thoracoabdominal Aortic Aneurysm, Ruptured
350	441.7	Thoracoabdominal Aortic Aneurysm, without Mention of Rupture
351	441.9	Aortic Aneurysm of Unspecified Site without Mention of Rupture
352	442.9	Aneurysm of Unspecified Artery Site
353	443.29	Dissection of Other Artery
354	443.9	Peripheral Vascular Disease, Unspecified
355	444.1	Embolism and Thrombosis of Thoracic Aorta
356	444.22	Arterial Embolism and Thrombosis of Lower Extremity
357	447.2	Rupture of Artery

358	447.71	Thoracic Aortic Ectasia
359	447.8	Other Specified Disorders of Arteries and Arterioles
360	458	Hypotension
361	458.0	Orthostatic Hypotension
362	458.1	Chronic Hypotension
363	458.29	Other Iatrogenic Hypotension
364	458.8	Other Specified Hypotension
365	458.9	Hypotension, Unspecified
366	459.89	Other Specified Circulatory System Disorders
367	459.9	Unspecified Circulatory System Disorder
368	557.0	Acute Vascular Insufficiency of Intestine
369	648.51	Congenital Cardiovascular Disorders Complicating Pregnancy, Childbirth, or the Puerperium, Delivered, with or without Mention of Antepartum Condition
370	648.61	Other Cardiovascular Diseases Complicating Pregnancy, Childbirth, or the Puerperium, Delivered, with or without Mention of Antepartum Condition
371	648.63	Other Cardiovascular Diseases Complicating Pregnancy, Childbirth, or the Puerperium, Antepartum Condition or Complication
372	674.51	Peripartum Cardiomyopathy, Delivered, with or without Mention of Antepartum Condition
373	674.54	Peripartum Cardiomyopathy, Postpartum Condition or Complication
374	745.0	Common Truncus
375	745.10	Complete Transposition of Great Vessels
376	745.11	Double Outlet Right Ventricle
377	745.2	Tetralogy of Fallot
378	745.3	Common Ventricle
379	745.4	Ventricular Septal Defect
380	745.5	Ostium Secundum Type Atrial Septal Defect
381	745.69	Other Endocardial Cushion Defects
382	746.01	Atresia of Pulmonary Valve, Congenital
383	746.09	Other Congenital Anomalies of Pulmonary Valve
384	746.3	Congenital Stenosis of Aortic Valve
385	746.4	Congenital Insufficiency of Aortic Valve
386	746.81	Subaortic Stenosis, Congenital
387	746.82	Cor Triatriatum
388	746.83	Infundibular Pulmonic Stenosis, Congenital
389	746.85	Coronary Artery Anomaly, Congenital
390	746.86	Congenital Heart Block
391	746.87	Malposition of Heart and Cardiac Apex
392	746.89	Other Specified Congenital Anomalies of Heart
393	746.9	Unspecified Congenital Anomaly of Heart
394	747.0	Patent Ductus Arteriosus
395	747.10	Coarctation of Aorta (Preductal) (Postductal)
396	747.20	Congenital Anomaly of Aorta, Unspecified
397	747.21	Congenital Anomalies of Aortic Arch
398	747.22	Congenital Atresia and Stenosis of Aorta
399	747.29	Other Congenital Anomalies of Aorta
400	747.3	Anomalies of Pulmonary Artery, Congenital
401	747.31	Pulmonary Artery Coarctation and Atresia
402	747.32	Pulmonary Arteriovenous Malformation

403	747.39	Other Anomalies of Pulmonary Artery and Pulmonary Circulation
404	747.42	Partial Anomalous Pulmonary Venous Connection
405	747.49	Other Anomalies of Great Veins
406	747.60	Congenital Anomaly of the Peripheral Vascular System, Unspecified Site
407	747.63	Congenital Anomaly of Upper Limb Vessel
408	747.69	Congenital Anomalies of Other Specified Sites of Peripheral Vascular System
409	747.81	Anomalies of Cerebrovascular System, Congenital
410	747.82	Congenital Anomaly of Spinal Vessel
411	747.89	Other Specified Congenital Anomalies of Circulatory System
412	747.9	Unspecified Congenital Anomaly of Circulatory System
413	782.3	Edema
414	785.0	Tachycardia, Unspecified
415	785.1	Palpitations
416	785.2	Undiagnosed Cardiac Murmurs
417	785.3	Other Abnormal Heart Sounds
418	785.5	Shock without Mention of Trauma
419	785.50	Shock, Unspecified
420	785.51	Cardiogenic Shock
421	785.59	Other Shock without Mention of Trauma
422	785.9	Other Symptoms Involving Cardiovascular System
423	786.5	Chest Pain
424	786.50	Unspecified Chest Pain
425	786.51	Precordial Pain
426	786.59	Other Chest Pain
427	794.30	Nonspecific Abnormal Function Study, Cardiovascular, Unspecified
428	794.31	Nonspecific Abnormal Electrocardiogram [ECG] [ekg]
429	794.39	Other Nonspecific Abnormal Function Study of Cardiovascular System
430	796.3	Nonspecific Low Blood Pressure Reading
431	861.01	Contusion of Heart without Mention of Open Wound Into Thorax
432	970.81	Poisoning by Cocaine
433	972.0	Poisoning by Cardiac Rhythm Regulators
434	972.4	Poisoning by Coronary Vasodilators
435	972.6	Poisoning by Other Antihypertensive Agents
436	972.9	Poisoning by Other and Unspecified Agents Primarily Affecting the Cardiovascular System
437	974.4	Poisoning by Other Diuretics
438	995.4	Shock Due to Anesthesia, Not Elsewhere Classified
439	996.00	Mechanical Complications of Unspecified Cardiac Device, Implant, and Graft
440	996.01	Mechanical Complication Due to Cardiac Pacemaker (Electrode)
441	996.02	Mechanical Complication Due to Heart Valve Prosthesis
442	996.03	Mechanical Complication Due to Coronary Bypass Graft
443	996.04	Mechanical Complication Due to Automatic Implantable Cardiac Defibrillator
444	996.09	Other Mechanical Complication of Cardiac Device, Implant, and Graft
445	996.1	Mechanical Complication of Other Vascular Device, Implant, and Graft
446	996.61	Infection and Inflammatory Reaction Due to Cardiac Device, Implant, and Graft
447	996.71	Other Complications Due to Heart Valve Prosthesis

448	996.72	Other Complications Due to Other Cardiac Device, Implant, and Graft
449	996.74	Other Complications Due to Other Vascular Device, Implant, and Graft
450	996.83	Complications of Transplanted Heart
451	997.1	Cardiac Complications, Not Elsewhere Classified
452	998.00	Postoperative Shock, Unspecified
453	998.01	Postoperative Shock, Cardiogenic
454	998.09	Postoperative Shock, Other
455	998.11	Hemorrhage Complicating a Procedure
456	V12.53	Personal History of Sudden Cardiac Arrest
457	V12.59	Other Personal History of Diseases of Circulatory System
458	V13.65	Personal History of (Corrected) Congenital Malformations of Heart and Circulatory System
459	V15.1	Personal History of Surgery to Heart and Great Vessels, Presenting Hazards to Health
460	V17.49	Family History of Other Cardiovascular Diseases
461	V42.1	Heart Transplant
462	V43.3	Heart Valve Replaced by Other Means
463	V45.01	Cardiac Pacemaker in Situ
464	V45.02	Automatic Implantable Cardiac Defibrillator in Situ
465	V45.81	Postsurgical Aortocoronary Bypass Status
466	V45.82	Percutaneous Transluminal Coronary Angioplasty, Postsurgical Status
467	V53.31	Fitting and Adjustment of Cardiac Pacemaker
468	V53.32	Fitting and Adjustment of Automatic Implantable Cardiac Defibrillator
469	V53.39	Fitting and Adjustment of Other Cardiac Device
470	V58.81	Encounter for Fitting and Adjustment of Vascular Catheter
471	V71.7	Observation for Suspected Cardiovascular Disease
472	V72.81	Preoperative Cardiovascular Examination
473	V81.2	Screening for Other and Unspecified Cardiovascular Conditions

Abbreviations: ICD-9, International Classification of Diseases, Ninth Edition

Supplemental Table 2: Selective Cardiac Diagnoses List with associated cardiac diagnosis categories

Number	ICD-9 Code	Diagnosis Description	ACS/CAD	HF/Shock	Arrest/Arrythmia	Valve disease	Aorta/PAD	Other	Peri-Procedural	No acute CV
1	074.21	Coxsackie Pericarditis						X		
2	112.81	Candidal Endocarditis						X		
3	164.1	Malignant Neoplasm of Heart						X		
4	212.7	Benign Neoplasm of Heart						X		
5	272.0	Pure Hypercholesterolemia								X
6	272.2	Mixed Hyperlipidemia								X
7	272.4	Other and Unspecified Hyperlipidemia								X
8	272.6	Lipodystrophy								X
9	272.7	Lipidoses								X
10	272.9	Unspecified Disorder of Lipoid Metabolism								X
11	276.6	Fluid Overload		X						
12	337.01	Carotid Sinus Syndrome						X		
13	391.0	Acute Rheumatic Pericarditis						X		
14	391.1	Acute Rheumatic Endocarditis				X				
15	391.8	Other Acute Rheumatic Heart Disease				X				
16	394	Diseases of Mitral Valve				X				
17	394.0	Mitral Stenosis				X				
18	394.1	Rheumatic Mitral Insufficiency				X				
19	394.2	Mitral Stenosis with Insufficiency				X				
20	394.9	Other and Unspecified Mitral Valve Diseases				X				
21	395.0	Rheumatic Aortic Stenosis				X				
22	395.1	Rheumatic Aortic Insufficiency				X				
23	395.2	Rheumatic Aortic Stenosis with Insufficiency				X				
24	396.0	Mitral Valve Stenosis and Aortic Valve Stenosis				X				
25	396.1	Mitral Valve Stenosis and Aortic Valve Insufficiency				X				
26	396.2	Mitral Valve Insufficiency and Aortic Valve Stenosis				X				
27	396.3	Mitral Valve Insufficiency and Aortic Valve Insufficiency				X				
28	396.8	Multiple Involvement of Mitral and Aortic Valves				X				
29	396.9	Mitral and Aortic Valve Diseases, Unspecified				X				
30	397.0	Diseases of Tricuspid Valve				X				
31	398.90	Rheumatic Heart Disease, Unspecified				X				
32	398.91	Rheumatic Heart Failure (Congestive)		X						
33	401.0	Malignant Essential Hypertension						X		
34	402.00	Hypertensive Heart Disease, Malignant, without Heart Failure						X		
35	402.01	Hypertensive Heart Disease, Malignant, with Heart Failure		X						
36	402.10	Hypertensive Heart Disease, Benign, without Heart Failure						X		
37	402.11	Hypertensive Heart Disease, Benign, with Heart Failure		X						
38	402.90	Hypertensive Heart Disease, Unspecified, without Heart Failure						X		
39	402.91	Hypertensive Heart Disease, Unspecified, with Heart Failure		X						
40	404.00	Hypertensive Heart and Chronic Kidney Disease, Malignant, without Heart Failure and with Chronic Kidney Disease Stage I Through Stage IV, or Unspecified						X		
41	404.02	Hypertensive Heart and Chronic Kidney Disease, Malignant, without Heart Failure and with Chronic Kidney Disease Stage V or End Stage Renal Disease						X		
42	404.11	Hypertensive Heart and Chronic Kidney Disease, Benign, with Heart Failure and with Chronic Kidney Disease Stage I Through Stage IV, or Unspecified		X						
43	404.12	Hypertensive Heart and Chronic Kidney Disease, Benign, without Heart Failure and with Chronic Kidney Disease Stage V or End Stage Renal Disease						X		
44	404.13	Hypertensive Heart and Chronic Kidney Disease, Benign, with Heart Failure and Chronic Kidney Disease Stage V or End Stage Renal Disease		X						
45	404.90	Hypertensive Heart and Chronic Kidney Disease, Unspecified, without Heart Failure and with Chronic Kidney Disease Stage I Through Stage IV, or Unspecified						X		
46	404.92	Hypertensive Heart and Chronic Kidney Disease, Unspecified, without Heart Failure and with Chronic Kidney Disease Stage V or End Stage Renal Disease						X		
47	405.01	Malignant Renovascular Hypertension						X		
48	410	Acute Myocardial Infarction	X							
49	410.0	Acute Myocardial Infarction, of Anterolateral Wall	X							
50	410.00	Acute Myocardial Infarction, of Anterolateral Wall, Episode of Care Unspecified	X							
51	410.01	Acute Myocardial Infarction, of Anterolateral Wall, Initial Episode of Care	X							
52	410.02	Acute Myocardial Infarction, of Anterolateral Wall, Subsequent Episode of Care	X							
53	410.10	Acute Myocardial Infarction, of Other Anterior Wall, Episode of Care Unspecified	X							
54	410.11	Acute Myocardial Infarction, of Other Anterior Wall, Initial Episode of Care	X							
55	410.12	Acute Myocardial Infarction, of Other Anterior Wall, Subsequent Episode of Care	X							
56	410.20	Acute Myocardial Infarction, of Inferolateral Wall, Episode of Care Unspecified	X							
57	410.21	Acute Myocardial Infarction, of Inferolateral Wall, Initial Episode of Care	X							
58	410.22	Acute Myocardial Infarction, of Inferolateral Wall, Subsequent Episode of Care	X							
59	410.3	Acute Myocardial Infarction, of Inferoposterior Wall	X							
60	410.30	Acute Myocardial Infarction, of Inferoposterior Wall, Episode of Care Unspecified	X							
61	410.31	Acute Myocardial Infarction, of Inferoposterior Wall, Initial Episode of Care	X							
62	410.32	Acute Myocardial Infarction, of Inferoposterior Wall, Subsequent Episode of Care	X							
63	410.4	Acute Myocardial Infarction, of Other Inferior Wall	X							
64	410.40	Acute Myocardial Infarction, of Other Inferior Wall, Episode of Care Unspecified	X							
65	410.41	Acute Myocardial Infarction, of Other Inferior Wall, Initial Episode of Care	X							
66	410.42	Acute Myocardial Infarction, of Other Inferior Wall, Subsequent Episode of Care	X							
67	410.50	Acute Myocardial Infarction, of Other Lateral Wall, Episode of Care Unspecified	X							
68	410.51	Acute Myocardial Infarction, of Other Lateral Wall, Initial Episode of Care	X							
69	410.61	Acute Myocardial Infarction, True Posterior Wall Infarction, Initial Episode of Care	X							

70	410.7	Acute Myocardial Infarction, Subendocardial Infarction	X					
71	410.70	Acute Myocardial Infarction, Subendocardial Infarction, Episode of Care Unspecified	X					
72	410.71	Acute Myocardial Infarction, Subendocardial Infarction, Initial Episode of Care	X					
73	410.72	Acute Myocardial Infarction, Subendocardial Infarction, Subsequent Episode of Care	X					
74	410.80	Acute Myocardial Infarction, of Other Specified Sites, Episode of Care Unspecified	X					
75	410.81	Acute Myocardial Infarction, of Other Specified Sites, Initial Episode of Care	X					
76	410.82	Acute Myocardial Infarction, of Other Specified Sites, Subsequent Episode of Care	X					
77	410.9	Acute Myocardial Infarction, Unspecified Site	X					
78	410.90	Acute Myocardial Infarction, Unspecified Site, Episode of Care Unspecified	X					
79	410.91	Acute Myocardial Infarction, Unspecified Site, Initial Episode of Care	X					
80	410.92	Acute Myocardial Infarction, Unspecified Site, Subsequent Episode of Care	X					
81	411.0	Postmyocardial Infarction Syndrome	X					
82	411.1	Intermediate Coronary Syndrome	X					
83	411.81	Acute Coronary Occlusion without Myocardial Infarction	X					
84	411.89	Other Acute and Subacute Forms of Ischemic Heart Disease	X					
85	412	Old Myocardial Infarction						X
86	413	Angina Pectoris	X					
87	413.0	Angina Decubitus	X					
88	413.1	Prinzmetal Angina	X					
89	413.9	Other and Unspecified Angina Pectoris	X					
90	414.0	Coronary Atherosclerosis	X					
91	414.00	Coronary Atherosclerosis of Unspecified Type of Vessel, Native or Graft	X					
92	414.01	Coronary Atherosclerosis of Native Coronary Artery	X					
93	414.02	Coronary Atherosclerosis of Autologous Vein Bypass Graft	X					
94	414.04	Coronary Atherosclerosis of Artery Bypass Graft	X					
95	414.05	Coronary Atherosclerosis of Unspecified Type of Bypass Graft	X					
96	414.06	Coronary Atherosclerosis of Native Coronary Artery of Transplanted Heart	X					
97	414.10	Aneurysm of Heart (Wall)					X	
98	414.11	Aneurysm of Coronary Vessels	X					
99	414.12	Dissection of Coronary Artery	X					
100	414.19	Other Aneurysm of Heart					X	
101	414.2	Chronic Total Occlusion of Coronary Artery						X
102	414.4	Coronary Atherosclerosis Due to Calcified Coronary Lesion	X					
103	414.8	Other Specified Forms of Chronic Ischemic Heart Disease						X
104	414.9	Chronic Ischemic Heart Disease, Unspecified						X
105	415.0	Acute Cor Pulmonale		X				
106	416.0	Primary Pulmonary Hypertension		X				
107	416.9	Chronic Pulmonary Heart Disease, Unspecified						X
108	417.1	Aneurysm of Pulmonary Artery					X	
109	420	Acute Pericarditis					X	
110	420.90	Acute Pericarditis, Unspecified					X	
111	421.0	Acute and Subacute Bacterial Endocarditis				X		
112	421.9	Acute Endocarditis, Unspecified				X		
113	422.90	Acute Myocarditis, Unspecified		X				
114	422.91	Idiopathic Myocarditis		X				
115	422.99	Other Acute Myocarditis		X				
116	423.0	Hemopericardium					X	
117	423.1	Adhesive Pericarditis					X	
118	423.2	Constrictive Pericarditis					X	
119	423.3	Cardiac Tamponade					X	
120	423.8	Other Specified Diseases of Pericardium					X	
121	423.9	Unspecified Disease of Pericardium					X	
122	424.0	Mitral Valve Disorders				X		
123	424.1	Aortic Valve Disorders				X		
124	424.2	Tricuspid Valve Disorders, Specified as Nonrheumatic				X		
125	424.3	Pulmonary Valve Disorders				X		
126	424.90	Endocarditis, Valve Unspecified, Unspecified Cause				X		
127	424.91	Endocarditis in Diseases Classified Elsewhere				X		
128	425	Cardiomyopathy		X				
129	425.1	Hypertrophic Cardiomyopathy		X				
130	425.11	Hypertrophic Obstructive Cardiomyopathy		X				
131	425.18	Other Hypertrophic Cardiomyopathy		X				
132	425.3	Endocardial Fibroelastosis		X				
133	425.4	Other Primary Cardiomyopathies		X				
134	425.5	Alcoholic Cardiomyopathy		X				
135	425.8	Cardiomyopathy in Other Diseases Classified Elsewhere		X				
136	425.9	Secondary Cardiomyopathy, Unspecified		X				
137	426.0	Atrioventricular Block, Complete			X			
138	426.10	Atrioventricular Block, Unspecified			X			
139	426.11	First Degree Atrioventricular Block						X
140	426.12	Mobitz (Type) II Atrioventricular Block			X			
141	426.13	Other Second Degree Atrioventricular Block			X			

142	426.2	Left Bundle Branch Hemiblock							X
143	426.3	Other Left Bundle Branch Block							X
144	426.4	Right Bundle Branch Block							X
145	426.50	Bundle Branch Block, Unspecified							X
146	426.51	Right Bundle Branch Block and Left Posterior Fascicular Block							X
147	426.52	Right Bundle Branch Block and Left Anterior Fascicular Block							X
148	426.53	Other Bilateral Bundle Branch Block							X
149	426.54	Trifascicular Block							X
150	426.6	Other Heart Block			X				
151	426.7	Anomalous Atrioventricular Excitation			X				
152	426.82	Long Qt Syndrome			X				
153	426.89	Other Specified Conduction Disorders			X				
154	426.9	Conduction Disorder, Unspecified			X				
155	427.0	Paroxysmal Supraventricular Tachycardia			X				
156	427.1	Paroxysmal Ventricular Tachycardia			X				
157	427.2	Paroxysmal Tachycardia, Unspecified			X				
158	427.3	Atrial Fibrillation and Flutter			X				
159	427.31	Atrial Fibrillation			X				
160	427.32	Atrial Flutter			X				
161	427.41	Ventricular Fibrillation			X				
162	427.42	Ventricular Flutter			X				
163	427.5	Cardiac Arrest			X				
164	427.60	Premature Beats, Unspecified							X
165	427.61	Supraventricular Premature Beats							X
166	427.69	Other Premature Beats							X
167	427.81	Sinoatrial Node Dysfunction			X				
168	427.89	Other Specified Cardiac Dysrhythmias			X				
169	427.9	Cardiac Dysrhythmia, Unspecified			X				
170	428	Heart Failure		X					
171	428.0	Congestive Heart Failure, Unspecified		X					
172	428.1	Left Heart Failure		X					
173	428.2	Systolic Heart Failure		X					
174	428.20	Unspecified Systolic Heart Failure		X					
175	428.21	Acute Systolic Heart Failure		X					
176	428.22	Chronic Systolic Heart Failure							X
177	428.23	Acute on Chronic Systolic Heart Failure		X					
178	428.3	Diastolic Heart Failure		X					
179	428.30	Unspecified Diastolic Heart Failure		X					
180	428.31	Acute Diastolic Heart Failure		X					
181	428.32	Chronic Diastolic Heart Failure							X
182	428.33	Acute on Chronic Diastolic Heart Failure		X					
183	428.40	Unspecified Combined Systolic and Diastolic Heart Failure		X					
184	428.41	Acute Combined Systolic and Diastolic Heart Failure		X					
185	428.42	Chronic Combined Systolic and Diastolic Heart Failure							X
186	428.43	Acute on Chronic Combined Systolic and Diastolic Heart Failure		X					
187	428.9	Heart Failure, Unspecified		X					
188	429.0	Myocarditis, Unspecified		X					
189	429.1	Myocardial Degeneration		X					
190	429.2	Cardiovascular Disease, Unspecified		X					
191	429.3	Cardiomegaly		X					
192	429.4	Functional Disturbances Following Cardiac Surgery						X	
193	429.5	Rupture of Chordae Tendineae	X						
194	429.6	Rupture of Papillary Muscle	X						
195	429.71	Cardiac Septal Defect, Sequela of Myocardial Infarction	X						
196	429.79	Other Sequelae of Myocardial Infarction, Not Elsewhere Classified	X						
197	429.83	Takotsubo Syndrome		X					
198	429.89	Other Ill-Defined Heart Diseases					X		
199	429.9	Heart Disease, Unspecified					X		
200	440	Atherosclerosis	X						
201	440.0	Atherosclerosis of Aorta				X			
202	440.20	Atherosclerosis of Native Arteries of the Extremities, Unspecified				X			
203	440.21	Atherosclerosis of Native Arteries of the Extremities with Intermittent Claudication				X			
204	440.22	Atherosclerosis of the Extremities with Rest Pain				X			
205	440.23	Atherosclerosis of Native Arteries of the Extremities with Ulceration				X			
206	440.24	Atherosclerosis of Native Arteries of the Extremities with Gangrene				X			
207	440.30	Atherosclerosis of Bypass Graft of the Extremities, Unspecified Graft				X			
208	440.31	Atherosclerosis of Autologous Vein Bypass Graft of the Extremities				X			
209	440.32	Atherosclerosis of Nonautologous Biological Bypass Graft of the Extremities				X			
210	440.8	Atherosclerosis of Other Specified Arteries				X			
211	441.00	Dissecting Aneurysm of Aorta, Unspecified Site				X			
212	441.01	Dissecting Aneurysm of Thoracic Aorta				X			
213	441.03	Dissecting Aneurysm of Thoracoabdominal Aorta				X			

214	441.1	Thoracic Aortic Aneurysm, Ruptured				X			
215	441.2	Thoracic Aortic Aneurysm without Mention of Rupture				X			
216	441.3	Abdominal Aortic Aneurysm, Ruptured				X			
217	441.4	Abdominal Aortic Aneurysm without Mention of Rupture				X			
218	441.5	Aortic Aneurysm of Unspecified Site, Ruptured				X			
219	441.6	Thoracoabdominal Aortic Aneurysm, Ruptured				X			
220	441.7	Thoracoabdominal Aortic Aneurysm, without Mention of Rupture				X			
221	441.9	Aortic Aneurysm of Unspecified Site without Mention of Rupture				X			
222	442.9	Aneurysm of Unspecified Artery Site				X			
223	443.29	Dissection of Other Artery				X			
224	443.9	Peripheral Vascular Disease, Unspecified				X			
225	444.1	Embolism and Thrombosis of Thoracic Aorta				X			
226	444.22	Arterial Embolism and Thrombosis of Lower Extremity				X			
227	447.2	Rupture of Artery				X			
228	447.71	Thoracic Aortic Ectasia							X
229	447.8	Other Specified Disorders of Arteries and Arterioles				X			
230	458	Hypotension					X		
231	458.0	Orthostatic Hypotension					X		
232	458.1	Chronic Hypotension							X
233	458.29	Other Iatrogenic Hypotension						X	
234	458.8	Other Specified Hypotension					X		
235	458.9	Hypotension, Unspecified					X		
236	459.89	Other Specified Circulatory System Disorders					X		
237	459.9	Unspecified Circulatory System Disorder					X		
238	557.0	Acute Vascular Insufficiency of Intestine					X		
239	648.51	Congenital Cardiovascular Disorders Complicating Pregnancy, Childbirth, or the Puerperium, Delivered, with or without Mention of Antepartum Condition					X		
240	648.61	Other Cardiovascular Diseases Complicating Pregnancy, Childbirth, or the Puerperium, Delivered, with or without Mention of Antepartum Condition					X		
241	648.63	Other Cardiovascular Diseases Complicating Pregnancy, Childbirth, or the Puerperium, Antepartum Condition or Complication					X		
242	674.51	Peripartum Cardiomyopathy, Delivered, with or without Mention of Antepartum Condition	X						
243	674.54	Peripartum Cardiomyopathy, Postpartum Condition or Complication	X						
244	745.0	Common Truncus					X		
245	745.10	Complete Transposition of Great Vessels					X		
246	745.11	Double Outlet Right Ventricle					X		
247	745.2	Tetralogy of Fallot					X		
248	745.3	Common Ventricle					X		
249	745.4	Ventricular Septal Defect					X		
250	745.5	Ostium Secundum Type Atrial Septal Defect					X		
251	745.69	Other Endocardial Cushion Defects					X		
252	746.01	Atresia of Pulmonary Valve, Congenital			X				
253	746.09	Other Congenital Anomalies of Pulmonary Valve			X				
254	746.3	Congenital Stenosis of Aortic Valve			X				
255	746.4	Congenital Insufficiency of Aortic Valve			X				
256	746.81	Subaortic Stenosis, Congenital			X				
257	746.82	Cor Triatriatum					X		
258	746.83	Infundibular Pulmonic Stenosis, Congenital			X				
259	746.85	Coronary Artery Anomaly, Congenital					X		
260	746.86	Congenital Heart Block		X					
261	746.87	Malposition of Heart and Cardiac Apex					X		
262	746.89	Other Specified Congenital Anomalies of Heart					X		
263	746.9	Unspecified Congenital Anomaly of Heart					X		
264	747.0	Patent Ductus Arteriosus					X		
265	747.10	Coarctation of Aorta (Preductal) (Postductal)				X			
266	747.20	Congenital Anomaly of Aorta, Unspecified				X			
267	747.21	Congenital Anomalies of Aortic Arch				X			
268	747.22	Congenital Atresia and Stenosis of Aorta				X			
269	747.29	Other Congenital Anomalies of Aorta				X			
270	747.3	Anomalies of Pulmonary Artery, Congenital					X		
271	747.31	Pulmonary Artery Coarctation and Atresia					X		
272	747.32	Pulmonary Arteriovenous Malformation					X		
273	747.39	Other Anomalies of Pulmonary Artery and Pulmonary Circulation					X		
274	747.42	Partial Anomalous Pulmonary Venous Connection					X		
275	747.49	Other Anomalies of Great Veins					X		
276	747.60	Congenital Anomaly of the Peripheral Vascular System, Unspecified Site				X			
277	747.63	Congenital Anomaly of Upper Limb Vessel				X			
278	747.69	Congenital Anomalies of Other Specified Sites of Peripheral Vascular System				X			
279	747.81	Anomalies of Cerebrovascular System, Congenital					X		
280	747.82	Congenital Anomaly of Spinal Vessel					X		
281	747.89	Other Specified Congenital Anomalies of Circulatory System					X		
282	747.9	Unspecified Congenital Anomaly of Circulatory System					X		
283	782.3	Edema	X						
284	785.0	Tachycardia, Unspecified		X					
285	785.1	Palpitations					X		

286	785.2	Undiagnosed Cardiac Murmurs			X			
287	785.3	Other Abnormal Heart Sounds				X		
288	785.5	Shock without Mention of Trauma		X				
289	785.50	Shock, Unspecified		X				
290	785.51	Cardiogenic Shock		X				
291	785.59	Other Shock without Mention of Trauma		X				
292	785.9	Other Symptoms Involving Cardiovascular System				X		
293	786.5	Chest Pain	X					
294	786.50	Unspecified Chest Pain	X					
295	786.51	Precordial Pain	X					
296	786.59	Other Chest Pain	X					
297	794.30	Nonspecific Abnormal Function Study, Cardiovascular, Unspecified				X		
298	794.31	Nonspecific Abnormal Electrocardiogram [ECG] [ekg]				X		
299	794.39	Other Nonspecific Abnormal Function Study of Cardiovascular System				X		
300	796.3	Nonspecific Low Blood Pressure Reading				X		
301	861.01	Contusion of Heart without Mention of Open Wound Into Thorax				X		
302	970.81	Poisoning by Cocaine				X		
303	972.0	Poisoning by Cardiac Rhythm Regulators				X		
304	972.4	Poisoning by Coronary Vasodilators				X		
305	972.6	Poisoning by Other Antihypertensive Agents				X		
306	972.9	Poisoning by Other and Unspecified Agents Primarily Affecting the Cardiovascular System				X		
307	974.4	Poisoning by Other Diuretics				X		
308	995.4	Shock Due to Anesthesia, Not Elsewhere Classified		X				
309	996.00	Mechanical Complications of Unspecified Cardiac Device, Implant, and Graft					X	
310	996.01	Mechanical Complication Due to Cardiac Pacemaker (Electrode)					X	
311	996.02	Mechanical Complication Due to Heart Valve Prosthesis					X	
312	996.03	Mechanical Complication Due to Coronary Bypass Graft					X	
313	996.04	Mechanical Complication Due to Automatic Implantable Cardiac Defibrillator					X	
314	996.09	Other Mechanical Complication of Cardiac Device, Implant, and Graft					X	
315	996.1	Mechanical Complication of Other Vascular Device, Implant, and Graft					X	
316	996.61	Infection and Inflammatory Reaction Due to Cardiac Device, Implant, and Graft					X	
317	996.71	Other Complications Due to Heart Valve Prosthesis					X	
318	996.72	Other Complications Due to Other Cardiac Device, Implant, and Graft					X	
319	996.74	Other Complications Due to Other Vascular Device, Implant, and Graft					X	
320	996.83	Complications of Transplanted Heart		X				
321	997.1	Cardiac Complications, Not Elsewhere Classified					X	
322	998.00	Postoperative Shock, Unspecified					X	
323	998.01	Postoperative Shock, Cardiogenic		X				
324	998.09	Postoperative Shock, Other					X	
325	998.11	Hemorrhage Complicating a Procedure					X	
326	V12.53	Personal History of Sudden Cardiac Arrest						X
327	V12.59	Other Personal History of Diseases of Circulatory System						X
328	V13.65	Personal History of (Corrected) Congenital Malformations of Heart and Circulatory System						X
329	V15.1	Personal History of Surgery to Heart and Great Vessels, Presenting Hazards to Health						X
330	V17.49	Family History of Other Cardiovascular Diseases						X
331	V42.1	Heart Transplant		X				
332	V43.3	Heart Valve Replaced by Other Means			X			
333	V45.01	Cardiac Pacemaker in Situ					X	
334	V45.02	Automatic Implantable Cardiac Defibrillator in Situ						X
335	V45.81	Postsurgical Aortocoronary Bypass Status					X	
336	V45.82	Percutaneous Transluminal Coronary Angioplasty, Postsurgical Status					X	
337	V53.31	Fitting and Adjustment of Cardiac Pacemaker					X	
338	V53.32	Fitting and Adjustment of Automatic Implantable Cardiac Defibrillator					X	
339	V53.39	Fitting and Adjustment of Other Cardiac Device					X	
340	V58.81	Encounter for Fitting and Adjustment of Vascular Catheter					X	
341	V71.7	Observation for Suspected Cardiovascular Disease				X		
342	V72.81	Preoperative Cardiovascular Examination						X
343	V81.2	Screening for Other and Unspecified Cardiovascular Conditions						X

Abbreviations: ICD-9, International Classification of Diseases, Ninth Edition; ACS, acute coronary syndrome; CAD, coronary artery disease; HF, heart failure; PAD, peripheral arterial disease; CV, cardiovascular.

Supplemental Table 3: ICU care indication suggested by principal diagnosis codes

Principal diagnosis codes	Category	ICD9-codes
Pulmonary disease	Pulmonary	460-519
Diseases of pulmonary circulation	Pulmonary	415-417
Infectious diseases	Infectious Diseases/sepsis	001-139
Metabolic disorder	Renal/Metabolic/Toxic	240-279
Renal insufficiency	Renal/Metabolic/Toxic	580-629
Poisoning	Renal/Metabolic/Toxic	960-989
Digestive disease	Digestive system	520-579
Diseases of the musculoskeletal system & connective tissue	Skin/Soft tissue/Musculoskeletal	710-739
Diseases of the skin and subcutaneous tissue	Skin/Soft tissue/Musculoskeletal	680-709
Other forms of heart disease	Cardiovascular	420-429
Cerebrovascular disease	Neurologic	430-438
Neurologic disease	Neurologic	320-389
Mental disorders	Neurologic	290-319
Ischemic heart disease	Cardiovascular	410 - 414
Arteries and veins	Cardiovascular	440-459
Hypertensive disease	Cardiovascular	401-405
Chronic rheumatic heart disease	Cardiovascular	393-398
Acute Rheumatic fever	Cardiovascular	390-392
Trauma	Trauma/Procedural complication	800-959
Complications peculiar to certain specified procedures	Trauma/Procedural complication	996
Neoplasms	Heme-oncology	140-239
Diseases of the blood and blood-forming organs	Heme-oncology	280-289
Supplementary classification of factors influencing health status and contact with health services	Other/unspecified	V01-V86
Congenital anomalies	Other/unspecified	740-759
Symptoms, signs, and ill-defined conditions	Other/unspecified	780-799
Other complications of procedures, NEC	Other/unspecified	998
Complications affecting specified body systems, not elsewhere classified	Other/unspecified	997
Complications of pregnancy, childbirth, and the puerperium	Other/unspecified	630-677
Complications of medical care, not elsewhere classified	Other/unspecified	999
Other and unspecified effects of external causes	Other/unspecified	990-995
Heart failure	Cardiovascular	428

Source: Frequency of all ICD-9 codes for adult ICU-related hospital admissions [Internet]. Available from: https://physionet.org/mimic2/UserGuide/node86.html

Supplemental Table 4: Odds Ratio Estimates For Model 3

Variable Comparisons or Unit Increase	OR	95% Confidence Limits		P value
Admission to Non-CICU vs CICU	0.863	0.757	0.984	0.0276
Unit increase in age (years)*	1.034	1.029	1.040	<0.0001
Unit increase in SOFA score*	1.265	1.244	1.287	<0.0001
Unit increase in packs-per-day tobacco (packs)*	0.903	0.759	1.074	0.2493
Unit increase in Elixhauser score*	1.010	0.982	1.040	0.4851
Male vs female	0.841	0.747	0.947	0.0041
Payer: Medicaid vs Medicare	1.467	1.094	1.969	0.0086
Payer: Other insurance vs Medicare	1.107	0.906	1.353	
Payer: Self-insured vs Medicare	1.683	1.128	2.511	
Payer: Unknown vs Medicare	1.289	1.026	1.619	
Race: Caucasian vs African American	0.907	0.752	1.093	0.1310
Race: Other/Specified vs African American	1.440	0.935	2.218	
Race: Unknown/Unspecified vs African American	0.945	0.733	1.218	
Admit year 2010 vs 2009	1.074	0.834	1.382	0.0062
Admit year 2011 vs 2009	0.763	0.590	0.986	
Admit year 2012 vs 2009	0.765	0.592	0.989	
Admit year 2013 vs 2009	0.717	0.557	0.922	
Admit year 2014 vs 2009	0.864	0.650	1.149	
ACS/CAD vs "other" cardiac diagnoses	0.467	0.368	0.592	<0.0001
Aortic and PAD vs "other" cardiac diagnoses	0.476	0.352	0.646	
Cardiac arrest/Arrhythmia vs "other" cardiac diagnoses	1.154	0.906	1.472	
Heart failure/Shock vs "other" cardiac diagnoses	0.851	0.657	1.103	
Multiple cardiac diagnoses vs "other" cardiac diagnoses	0.753	0.571	0.992	
No acute cardiac diagnosis vs "other" cardiac diagnoses	0.628	0.404	0.978	
Peri-procedureal monitoring vs "other" cardiac diagnoses	0.553	0.356	0.858	
Valvular heart disease vs "other" cardiac diagnoses	0.224	0.157	0.318	

CICU level 1 vs 2	1.438	0.509	4.063	0.4930
Hospital bed size 200 - 499 vs ≥ 500	1.784	0.721	4.412	0.2101
Hospital setting Urban vs Rural	0.262	0.108	0.636	0.0031
Teaching hospital Yes vs No	1.061	0.380	2.962	0.9102
Annual admissions 10001-20000 vs ≤10000	1.466	0.613	3.507	0.1137
Annual admissions >20000 vs ≤10000	0.564	0.201	1.583	
Respiratory disease Yes vs No	2.834	2.461	3.263	<0.0001
Infectious disease Yes vs No	2.082	1.822	2.379	<0.0001
Renal/Metabolic disease Yes vs No	1.111	0.868	1.423	0.4017
Gastrointestinal disease Yes vs No	1.254	1.109	1.417	0.0003
Skin/Soft tissue/Muscle disease Yes vs No	0.876	0.766	1.001	0.0523
Neurologic disease Yes vs No	1.290	1.137	1.462	<0.0001
Traumatic disease/Procedural complication Yes vs No	1.044	0.878	1.242	0.6234
Hematologic/Oncologic disease Yes vs No	0.908	0.801	1.029	0.1315
Other/Unspecified disease Yes vs No	0.958	0.674	1.363	0.8129

*Effects of continuous variables are assessed as one unit change from the mean

P values displayed are for Type III Tests of Fixed Effects for each variable category

Abreviations: CICU, cardiac intensive care unit; SOFA, sequential organ failure assesment; ACS, acute coronary syndrome; CAD, coronary artery disease; PAD, peripheral arterial disease.

Supplemental Table 5: Mortality in Non-CICU vs CICU

Subgroup	N	Model 2 OR (95% CI)	Model 3 OR (95% CI)
No MCV	15346	1.12 (0.98-1.28)	0.89 (0.78-1.03)
No Pressors	12412	**1.36 (1.16-1.58)**	1.08 (0.92-1.26)
No MCV or Pressors	12141	**1.40 (1.20-1.64)**	1.10 (0.94-1.30)
Heart Failure/Shock	1370	1.37 (0.99-1.90)	1.16 (0.83-1.62)
Heart Failure/Shock without MCV or pressors	1057	**1.67 (1.13-2.48)**	1.36 (0.90-2.06)
ACS/CAD	7259	0.92 (0.71-1.19)	**0.71 (0.54-0.93)**
ACS/CAD without MCV or pressors	5959	1.35 (1.0-1.83)	1.07 (0.77-1.48)
Arrythmia/Arrest	2088	**1.34 (1.02-1.77)**	1.11 (0.83-1.49)
Arrythmia/Arrest without MCV or pressors	1473	**1.92 (1.30-2.83)**	1.41 (0.93-2.14)

Abbreviations: CICU, cardiac intensive care unit; OR, odds ratio;
CI, confidence interval; MCV, mechanical ventilation; ACS, acute coronary syndrome;
CAD, coronary artery disease
